# Exploring the Opinions of Irish Dairy Farmers Regarding Male Dairy Calves

**DOI:** 10.3389/fvets.2021.635565

**Published:** 2021-04-20

**Authors:** James W. Maher, AnneMarie Clarke, Andrew W. Byrne, Rob Doyle, Martin Blake, Damien Barrett

**Affiliations:** Department of Agriculture, Food and the Marine, Dublin, Ireland

**Keywords:** calf welfare, dairy farmers, male calves, qualitative research, Ireland

## Abstract

**Background:** There has been very little previous research in Ireland on the farmers' opinions regarding calf welfare issues. Calf welfare, particularly for male dairy calves, has assumed greater importance in Ireland in recent years due, in part, to an increase in the number of dairy cattle over the past decade. The objective of this study was to explore dairy farmers' views on a broad range of issues related to the expansion in the dairy herd.

**Methods:** A survey was developed to capture the views of farmers regarding male dairy calves. The majority of questions were quantitative, and a final open-ended question collected qualitative data. The survey was distributed to ~2,900 dairy farmers via text message and 881 responses were received.

**Results:** The sample was composed almost entirely of dairy farmers, although ~20% also had a beef enterprise on their farm. Fifty eight percent of the farmers were concerned with the increase in the number of male dairy calves in recent years. The EU's abolition of milk quotas, the profitability of dairy farming compared to other farm types, and guidance from farm advisors were the three highest ranked drivers behind the increase in the number of male dairy calves. The three highest ranked options for managing the number of male dairy calves were to increase exports, encourage greater use of sexed semen, and improve the beef merit of these calves. Eighty five percent of respondents stated that individual farmers had responsibility for making changes to the number of male dairy calves. The main themes arising from analysis of the responses to the open-ended question, seeking any additional comments, were breed, beef price, live exports, and sexed semen.

**Conclusions:** Dairy farmers recognized the responsibility they have for making changes in respect of male dairy calves, and many demonstrated a willingness to make changes in this regard. The important role of other stakeholders, particularly suckler (system where reared from calf to beef) farmers, in rearing male dairy calves for beef production was also recognized. However, the issues of who bears the risks and costs associated with greater integration will have to be carefully considered.

## Introduction

Since 2015, with the abolition of milk quotas in the European Union, there has been considerable expansion in the Irish dairy industry (1). The Irish Department of Agriculture had, in anticipation of the abolition of quotas, targeted a 50% growth in milk production in the strategy document Food Harvest 2020 (2), which a range of key stakeholders from across the agricultural sector helped inform. Regarding the increase in the national herd, the number of dairy cows in Ireland increased by 27% between 2013 and 2018 (3). As a consequence of the increased dairy cow numbers, there are increased numbers of male and female dairy bred beef animals coming onto the market. The issue of how to manage male dairy calves has been controversial in several countries, particularly where these young animals are euthanised, and has received prominent media attention (4).

Looking comparatively at how other countries manage male dairy calves, New Zealand and Australia do not have well-established industries for raising these calves, leading to the majority being transported long distances to be slaughtered within days of birth (5). For example, the significant increase in New Zealand dairy production since 1990 (6) led to a focus on traits for dairy productivity. This resulted in the male progeny from such cows, particularly Jersey and Jersey/Holstein-Friesian cross, having inferior beef characteristics, with the majority of these calves being slaughtered (7). The Irish dairy industry has modeled itself on New Zealand's pasture-based production system (8). In Europe and North America, the majority of male dairy calves contribute to the red meat industry (9), but the move to New Zealand type genetics in Ireland has led to increased numbers of calves with inferior beef characteristics, which beef farmers have difficulty in making a reasonable economic margin on.

Ireland differs from most other European Union countries; most Irish dairy farms are pasture based with spring calving (10, 11). As a consequence, there is a seasonal surplus of male dairy calves born on Irish dairy farms each year. A significant outlet for these calves is export to continental Europe, where there are well-established veal industries (12). Maintaining access to these export markets is a priority for Irish farm organizations, in order to maintain competition in the domestic beef market. However, the issue of live exports is of concern to the EU, with an EU parliament committee currently looking at animal welfare during transport. Therefore, like in Australia and New Zealand, surplus male dairy calves are a challenge for the Irish dairy industry (13).

While the present study does not directly address the welfare of male dairy calves, the increased number of male dairy calves in Ireland has the potential to result in welfare issues over the coming years if these animals are not managed in an appropriate manner. Driessen (14) recognized how the voices of farmers have been largely absent from debates on animal welfare and environmental concerns. Before specific new policies are introduced it is important to include stakeholders who will be responsible for their implementation in the process (15). By seeking the views of Irish farmers, this study aims to include farmers in the policy development process, which is particularly important given the inclusion of actors in policy development can lead to greater welfare compliance (16). For example, in Ireland, when suckler farmers were included in designing welfare initiatives the resulting initiatives were more practical and relevant (17). Regarding specific themes that have emerged from researching farmers' views on animal welfare, Cornish et al. (18) described how farmers' perceptions of animal welfare can be grouped into two categories; those concerned with the physical health and productivity of animals (focus on achieving economic results) and those concerned by broader aspects of well-being including the ability of animals to express natural behaviors (focus on moral and ethical concerns). There have been several studies examining dairy farmer's perspectives on welfare of dairy cattle in other jurisdictions, for example Sumner et al. (19) in Canada, Wolf et al. (20) in USA, and Vetouli et al. (21) in Norway and Sweden (specifically regarding organic dairy calves, which connected good welfare with the concept of naturalness). The US study demonstrated how farmers believe they are the actors with the most influence on calf welfare, followed by veterinarians, while the Canadian study highlighted the importance of farmer-veterinarian interaction for dairy cattle welfare. However, relatively little is known about Irish farmers' perspectives on animal welfare as Irish farmers' views on animal welfare are rarely sought, even though farmers “are the ones actually able to improve animal welfare” (22). Ventura et al. (23) found that veterinary practitioners believe farmers are the most important stakeholder in the improvement of animal welfare. Despite the importance of the farmer's role in improving welfare, very few previous studies have sought Irish farmers' views on how male dairy calves should be managed and this study aims to fill a gap in the existing literature. Given this dearth of knowledge, the objective of this study was to gather information on the perspectives of Irish dairy farmers regarding the issue of male dairy calves and on their preferred potential policy responses to manage the number of male calves. The availability of farmers' views on these issues may help inform future Departmental strategy on the management of male dairy calves.

## Methods

### Survey Development

A self-completion survey using the *SurveyMonkey* software package was created by the One Health Scientific Support (OHSS) team within the Irish Department of Agriculture, Food and the Marine (DAFM), with expertise in social science, veterinary, epidemiology and animal science backgrounds. The topics chosen were based on the project proposal that the OHSS received from the Animal Welfare division of DAFM, which had set out the key issues the Animal Welfare division wanted examined. Input was also provided by senior official veterinarians with responsibility for animal health and welfare policies. This collaboration took place during face-to-face and remote meetings. Various drafts of the survey were circulated to the relevant stakeholders (veterinary and administrative staff within DAFM), and changes were made following constructive feedback.

### Survey Design

In total, there were nineteen questions to be answered. An information section was presented at the outset, assuring potential participants that their anonymity would be protected and that their participation was voluntary, and the first question then asked respondents if they consented to participate in the study. Seventeen quantitative questions, comprising multiple choice and ranking style questions, were included in the survey. Where multiple responses were available the order of the options was randomized to minimize responder bias. In the ranking style questions, the participants had to rank each of the options; with 1 being what they felt the most important factor was. The method used for scoring these responses was to assign a reverse score, i.e., in a question with seven options the most important factor, ranked by a farmer at number 1, was given a score of 7. The weighted average was then used to determine the rankings. It was made compulsory to provide a response to each question before one could progress to answering the next question. Section breaks were also used, in conjunction with automatic skipping, to bring respondents to the end of the survey when they had completed all questions of relevance to their demographic cohort.

The final question was an open-ended question, asking participants to provide any further thoughts they had in a free text field; “Please provide any additional comments or suggestions you may have in the box below.” Qualitative research asks participants “to describe their experiences in ways that are meaningful to them” (24). Although the findings of such research cannot be generalized to other contexts, this method helps provide a greater understanding of certain issues, through the unique perspective of the participants. While the current survey did not allow for the collection of qualitative data in the same way as an interview or focus group, our rationale for incorporating a free text question to this survey was to help improve DAFM's understanding of dairy farmers' opinions on these topics. The final question afforded farmers an opportunity for their voices to be heard in an unprompted manner and not in response to direct questioning. This coincides with the aim of this paper to include the views of farmers in policy development. A list of the questions asked, and available responses, is available in Additional File 1 in Supplementary Information.

### Data Collection

There are ~18,000 dairy farmers in Ireland (25). The OHSS team issued a link to the survey using text messages to a sample of ~ 2,900 farmers, derived from a nationwide database of dairy famers held by the organization Animal Health Ireland on January 21st, 2020. This method of distribution was chosen in order to reach a wide audience of dairy farmers from all around Ireland. In previous research links have been distributed to online surveys of Irish farmers, for example Meunier et al. (26). Two text message reminders were subsequently issued and, by the closing date of February 10th, 2020, a total of 881 responses had been received. This would suggest a response rate of ~30%, but it is not possible to determine an accurate response rate given the possibility that recipients of the text message may have forwarded it to friends and family to complete. Overall, 98.6% (869) of the 881 respondents consented to participate in the study.

### Data Analysis

SurveyMonkey aided the presentation of the results of the quantitative questions (Q2–Q18), generating tables and graphs presenting the results of each question; the output file is available in Additional File 2 in Supplementary Information. SurveyMonkey was used to generate rating scales for ranking style questions, as in previous research including Sayers et al. (27). Regarding the qualitative question, a total of 402 responses were received (representing 46.2% of the respondents who had consented to participate in the study). The first step in the analysis of these responses was to identify any unusable replies, for example, where the respondent had indicated that they had no further comment. Sixteen responses were removed at this stage, leaving 386 substantive responses for further analysis, amounting to a total word count of almost 13,000 words.

The next step was for the lead author to read through each of the individual responses and assign a tag, or multiple tags where appropriate, categorizing the responses as falling under specific themes. The coding was done manually, with the aid of Microsoft Excel. There were no *a priori* assumptions of what themes would arise. An inductive coding approach was used; the codes applied to the responses were single words or short phrases that represented the meaning behind the content of the response. Multiple codes could be applied to longer responses where several different issues were raised. The responses were categorized following a careful reading of all of the responses on multiple occasions and the responses were subsequently rechecked multiple times by the lead author to ensure that the correct codes had been applied to each response. The responses were then grouped by the themes that were coded in order to determine the most frequently mentioned topics.

There is evidently a strong subjective element to any such categorization exercise as judgment calls frequently have to be made in deciding what theme most accurately represents the content of the response (28). It is not claimed that the list of themes used to categorize the qualitative responses is an exhaustive one, rather the themes cover those topics which could be clearly defined, and which arose in multiple responses. One hundred and twelve of the responses referred, in whole or partially, to issues that could not be categorized clearly within these chosen themes. In this paper only the most common themes will be presented and discussed. Select, representative quotes will be used to demonstrate the attitudes of the dairy farmers. The principles governing the choice of quote were to use a quote that “is illustrative of the point the writer is making about the data, it is reasonably succinct, and it is representative of the patterns in data” (29).

## Quantitative Results

### Completion

Six hundred and seventy two respondents (78% of those who consented to participate) completed all seventeen quantitative questions.

### Demographics

As seen in Table 1, the vast majority of participants had a dairy element to their farm enterprise. Over 60% of the respondents were aged over 45 years. Approximately 90% of the respondents were from Munster and Leinster. Ninety four percent of participants identified as being full time farmers.

**Table 1:** Demographic results.

	**Frequency**	**%**
**FARM TYPE**		
Dairy	693	80.1
Beef	4	0.5
Dairy & Beef	163	18.8
Other	5	0.6
**AGE**		
18–24	23	2.7
25–34	79	9.1
35–44	233	26.9
45–54	283	32.7
55–64	191	22.1
65+	56	6.5
**PROVINCE**		
Connacht	36	4.2
Leinster	223	25.8
Munster	544	62.9
Ulster	62	7.2
**WORK PATTERN**		
Full-time	814	94.1
Part-time	51	5.9

### Attitudes to Number of Male Dairy Calves

Fifty eight percent of participants stated that they were concerned about the large number of male dairy calves in recent years. An interesting observation regarding this question was that 184 participants (21% of those who had consented to take part) dropped out of the survey at this juncture, without providing a response. Lack of engagement in studies of welfare issues has been observed previously. In Butler et al. (30), some potential participants believed that assessing the welfare of horses was a pointless exercise and refused to participate in the study.

Regarding potential drivers of the increase in number of male dairy calves, the abolition of milk quotas (5.71), the profitability of dairy farming compared to other farm types (5.37) and guidance from farm advisors (4.25) were the three highest ranked factors. DAFM strategy (2.82) was viewed to be the least important driver. Increasing calf exports (5.85), encouraging greater use of sexed semen (5.66), and improving beef merit (5.1) were identified as being the most effective options in managing the number of male dairy calves. The two least popular options were the reintroduction of quotas (3.11) and rearing these calves for beef on their own farms (3.49). The subsequent question asked farmers to rank their preferred policy options in the scenario that live exports were to cease. The ranked order of the policy options, aside from the now removed “increasing calf exports” option, remained the same; encouraging greater use of sexed semen (5.13), improving beef merit (4.62), and try to establish an Irish veal industry (4.55) were the three highest ranked options. Regarding responsibility for making changes to the number of male dairy calves, 85% of participants stated that individual farmers had responsibility in this area. Teagasc, a state agency responsible for agricultural research and advice (70%), and DAFM (51%) were the two organizations farmers identified as having most responsibility for making changes to the number of male dairy calves. Please see Additional File 2 for a more detailed breakdown of these results.

### Herd Statistics

#### Calf Accommodation

87.5% of farmers indicated that they had sufficient calf accommodation.

#### Number of Cows

The vast majority of the farmers had between 50 and 200 dairy cows on their farm (77%), with very few having <50 (8%) or more than 500 cows (2%).

#### Breed

Friesian (74%) and Holstein (60%) were the two most frequently used breeds on these dairy farms. Five percent of the farmers used pure bred Jersey cows, but a quarter had cross bred dairy cows in their herd, which included Jersey cross animals.

### Management of Male Dairy Calves

#### Purpose

Sixty eight percent of farmers believed that their male dairy calves are a product worthy of selling in their own right, while 32% of respondents felt that the main purpose of their male dairy calves was just to get the cow to produce milk.

#### Current Management

For this question, it was possible for farmers to select multiple options. Fifty one percent of the farmers sell their male dairy calves via a mart (live animal market), 49% sell directly to a dealer (a person who buys the animals and sells them immediately again to another client) or exporter (a person who buys animals to export them), while 24% rear the male calves for beef themselves. In respect of the 24% of farmers who selected the “other” option, most specified that they sold directly to other farmers. Only 1% of farmers indicated that they use contract rearing for their male dairy calves.

#### Willingness to Pay for Contract Rearing

Contract rearing refers to the practice whereby calves are moved to another farm for a different farmer to rear them in return for an agreed payment. The dairy farmers retain ownership and by using contract rearing they can free up labor, accommodation facilities, and grazing land, allowing them to focus on their dairy enterprise activities. Seventy percent disagreed with the statement “I am willing to pay for contract rearing for my male dairy calves,” indicating an unwillingness to pay for male calves to be contract reared.

Please see Additional File 2 for detailed statistical results of quantitative portion of survey.

## Qualitative Results

This section aims to present the results of the final, open-ended question of the survey. This question gave respondents a chance to voice any further opinions they had on the topics of calf welfare and male dairy calves. The responses were categorized into themes and the main themes emerging from the analysis are presented here.

### Breed (*n* = 84)

This theme covers responses that referred to the breed of cattle in general, or to specific named breeds. Regarding specific breeds, Jersey/Jersey cross cattle were mentioned the most often (*n* = 51). The issue of personal responsibility came up frequently in conjunction with breed; “*I think every farmer should be responsible for their own calves and if they want to cross breed (i.e. Jersey) they should not be making that calf someone else's problem*.” There was a perception that all dairy farmers are being tarnished by the negative views of Jersey/cross bred calves, best seen in the following quotes: (i) “*Dairy bull calf covers all breeds but the issue with the dairy bull is not with all breeds probably only one*”; (ii) “*Jersey and jersey cross calves have given all dairy calves a bad reputation*.” Solutions suggested by farmers included initiatives aimed at discouraging the use of these breeds, placing a levy on these animals, only using sexed semen for these breeds, ranging to severe outright bans on the use of Jersey artificial insemination straws.

Many farmers suggested other breeds as a better alternative for dairy farmers. Friesians/Holstein-Friesians (*n* = 18) were the second most commonly mentioned breeds. Specifically relating to the welfare of the animals, it was suggested that “*Friesian Holsteins have some chance of having some kind of life*.” Several farmers suggested that the issues with male dairy calves are a recent occurrence associated with a change in the breeds being used on dairy farms; “*When there was almost all British/Holstein Friesian cows there was no problem with bull calves from dairy herds*.” Some respondents suggested that dual purpose animals, which have value for both beef and dairy farmers, should be used instead, with Fleckvieh and Montbéliarde being mentioned in this regard.

### Beef Price (*n* = 57)

This theme covers responses where the price of beef received from factories was mentioned by farmers. The following quote is representative of the vast majority of these responses; “*The main problem is the price the factories are giving for quality beef*.” Some farmers claimed that if there was a reasonable price available at factories there would be no problem with male dairy calves. Farmers felt that a better price at the factory would act as an incentive for beef farmers to rear dairy calves for beef production; “*If the beef price was at a fair level (*€*4 per kg plus) there would be plenty of part time farmers who would be delighted to bring these cattle to finish*.” €4 per kilogram was mentioned several times as representing a fair price for beef.

### Sexed Semen (*n* = 53)

This theme covers responses where farmers mentioned the cost or efficacy of sexed semen. Farmers wrote about the need for better research into the use of sexed semen to improve conception rates and reduce cost. Given the higher cost associated with sexed semen several farmers suggested incentives should be offered by DAFM to make it more accessible. The need for a sexed semen lab in Ireland was also mentioned on numerous occasions; “*sexed semen should be produced here in Ireland to improve quality instead of going to the UK, with all that movement quality is comprised*.” The issue of using sexed semen was emphasized for farmers using Jersey/Jersey cross breeds “*Anyone using extreme milk genetics e.g. Jersey, should use sexed semen to reduce the volume of poor quality dairy males diluting the beef market*.”

### Live Exports (*n* = 51)

This theme covers responses where farmers spoke about the live export of male dairy calves from Ireland. Farmers stressed the importance of the continued availability of live exports as an outlet for dairy calves: “*All the talk of ban on exports is bad news for Ireland. We have to have export markets at calf level and older stock too otherwise there will be too many stock in the country*.” These farmers demonstrated an awareness of the consequences if live exports ceased to be option; “*If live exports go, we will have a very serious situation*.” Problems relating to capacity on ships and lairage abroad were raised as issues requiring serious governmental attention. The topic of exports was frequently mentioned in combination with references to demand for veal in other EU countries, particularly the Netherlands.

### Beef Merit (*n* = 49)

This theme covers responses where farmers mentioned the beef merit, or lack thereof, of animals coming from the dairy herd. Farmers recognized the importance of considering the beef merit of dairy calves in addition to their dairy traits; “*Every calf born must also be considered a beef animal as well as a dairy animal*.” The problems with the beef merit of Jersey/Jersey cross calves were raised; “*they lack beef qualities such as bigger carcasses and muscle production*”. Some farmers were willing to slightly reduce the productivity of their dairy enterprise by placing greater emphasis on beef traits; “*give more value to beef side of cow with perhaps a small sacrifice in milk solids output so produce male calves people want*.”

### Welfare (*n* = 44)

This theme covers responses where farmers mentioned animal welfare in general or specifically wrote about the welfare of male dairy calves. Most farmers who spoke about welfare expressed very strong sentiments in favor of protecting the welfare of all animals; “*livestock should not be treated simply as 'business inventory'. These are living and breathing animals and must be treated with the compassion and respect that they deserve*.” Farmers were supportive of very severe penalties if other farmers were found to be treating their animals poorly; “*Where actual animal welfare breaches occur they should be investigated and when breaches are detected they should be severely punished and published*.”

There were very few references to killing dairy calves, only thirteen were in favor of euthanasia, at least in certain circumstances: “*This study has not addressed the real elephant in the room. Bull calves are an unwanted by-product of dairying - they are relatively worthless. Why wasn't controlled, humane, culling at birth an option?”* Seven were against euthanasia in any circumstances “*Any person deliberately killing male beef calves should have their herd number cancelled by Dept of Agriculture*.”

### Organizations

#### DAFM (*n* = 32, Including References to Minister for Agriculture)

While there were a small number of negative comments aimed at DAFM, mainly regarding beef price, the majority of comments spoke about the role of DAFM in providing a solution to the issues regarding the large number of male dairy calves; “*Farmers desperately need the Dept of Ag to protect us from the mess*.” The need for DAFM to set out a clear, long-term dairy strategy was raised; “*the department have to produce a proper long term plan for the industry, currently farms are making long term investments, then suddenly the rules change and just because there is a grant we are expected to change over night*.” The roles of DAFM in maintaining export markets and monitoring calf welfare on farm were also mentioned.

#### Teagasc (*n* = 28)

There was a widespread perception that farmers are now suffering the consequences of the advice they had received; “*Teagasc encouraged expansion without considering male dairy calves. Now we have to deal with the consequences of crossbreeding jerseys*.” Advice from the cooperatives (*n* = 7) were also mentioned in some of these comments attempting to explain how the current situation arose. Many of the farmers who mentioned Teagasc identified Teagasc's responsibility for providing solutions going forward; “*Need Teagasc to drive research and knowledge transfer on rearing dairy bred beef* ”.

#### Bord Bia (*n* = 11)

Farmers referred to Bord Bia's crucial role in identifying new export markets and maintaining exist export markets.

### Farm Type (*n* = 17)

This theme covers responses which mentioned different farm enterprise types, particularly suckler farming. Dairy farmers spoke about the important role suckler farmers could have in rearing their male dairy calves, with some expressing a willingness to give these calves away for free. Farmers also felt that the beef from male dairy calves would be of similar quality to beef currently produced by suckler farmers; “*the male dairy calf is as good in terms of quality of meat as suckler counterparts*.” Coinciding with the results in the quantitative questions very few farmers spoke of the usefulness of contract rearing as a means of managing male dairy calves. Several responses referred to competition or potential conflict between dairy and beef farmers in Ireland; “*Don't force a profitable dairy sector subsidize a loss-making beef industry either by paying for contract rearing or forcing the use of beef straws*.”

### Other

A small number of farmers spoke about a range of other topics including, *inter alia*, the expansion in the dairy herd, the reintroduction of quotas, domestic veal production, environmental concerns, and media coverage. These will not be examined in this paper.

## Discussion

To the authors' knowledge, this is the first survey to study Irish dairy farmers' attitudes to the issue of male dairy calves. Selection of suitable sires, the more widespread use of sexed semen, live exports, and the integration of male dairy calves into beef finishing systems were found to be the main strategies in addressing the issue.

### Breed

While the most common theme arising in the free text responses related to breed, especially Jersey and Jersey cross cattle; the herd statistics of the cohort surveyed indicated that only a minority of dairy farms have these breeds in their herd. It is difficult to reconcile the difference between the perception of how significant a problem these breeds represent and the fact that they represent a small, declining, proportion of the national herd (31). The sustained focus on the issue in the agricultural media (32) and the poor beef characteristics of these breeds may help to explain why it arose so frequently in responses in this survey. This is not a uniquely Irish concern, with media, particularly online news sources, in New Zealand regularly raising the issue of male dairy calves (33, 34).

### Responsibility

A minority of farmers questioned the strategic direction of the industry and the advice provided to farmers by Teagasc and milk processors. Very few respondents mentioned the responsibility of organizations in making changes regarding male dairy calves. Rather, many farmers mentioned the role of farmers in bringing about changes. The link between personal responsibility and behavioral change has long been recognized in public policy (35). Furthermore, given Ventura et al. (23) recognized the central role of farmers in improving animal welfare, it was encouraging that, when asked to select all stakeholders responsible for the making changes to the number of male dairy calves, the most commonly selected response (by 85% of respondents) was the individual farmer. Dolan et al. [(36), p. 71] described how personal responsibility and government involvement in changing behavior are not mutually exclusive; “government may spark initial changes that lead to reinforcing behaviors that manifest personal responsibility.” This coincides with research literature on nudging behavior (37).

The interaction of breed and farmer responsibility in the free text responses was another interesting finding; the implication being that if farmers decide to have cross bred (particularly Jersey) animals in their dairy herd then they should also be responsible for managing any consequences of this decision. The concept of personal responsibility also arose in respect of beef merit with some farmers displaying a willingness to reduce dairy productivity in order to ensure that male calves from their herds would be able to be reared for beef. A small number of farmers also described how they would be willing to contribute toward the cost of establishing a sexed semen lab in Ireland. All of these findings serve to demonstrate the willingness of some Irish dairy farmers to accept personal responsibility and their openness to making changes which could ameliorate the situation. This will be crucial to the success of any future strategy, as the people responsible for implementing the strategy must be motivated to change (38). Dwane et al. (17) demonstrated that when the farmers were motivated to change their behavior, in that study using financial incentives, welfare improved and certain new welfare practices were expected to continue into the future. It is also worth acknowledging that, while farmers ranked themselves as the stakeholder most responsible for making changes to the number of male dairy calves, other administrative changes may be required to help and support farmers making changes to their behavior in this regard.

### Live Exports

The issue of live exports is somewhat contentious and has been the subject of recent discussions in the Irish Parliament (39). While farmers demonstrated an awareness of the possible consequences if live exports were to cease to be available as an outlet, very few farmers indicated an awareness that such a scenario could occur in the short to medium term. Risks to the continued availability of live exports due to welfare concerns or adverse media coverage causing reputational damage were not identified by many farmers. The possibility of a ban on live exports is a real one, given recent suggestions that live exports from England and Wales may be banned in what would be a first for European nations (40). A recent study from Wilson et al. (41) surveyed the existing research, which suggests a 12-h maximum transport time for young calves and that rest stops may provide little benefit; these findings will be problematic for Irish live exports given the destination for calves is continental Europe.

### Welfare

Much like Kauppinen et al. (22), the Irish farmers who mentioned welfare in their responses were against practices that would cause negative effects on an animal's welfare. The responses mentioning welfare did not just cover farmers who were critical of other farmers who abuse or mistreatment animals; a smaller number of farmers recognized the importance of actively treating animals well in terms of taking steps to ensure that male calves had proper housing and were well-fed before leaving their farm. This is indicative of empathy toward the animals in their herd, which Balzani and Hanlon (16) indentified as the foundation of farmers' opinions on animal welfare and their ability to meet the needs of their animals. The majority of responses were general in nature, condemning any mistreatment of animals; there were very few mentions of specific negative welfare behaviors or actions farmers could take to improve welfare. It is important to be aware of farmers' attitudes to welfare, given Dwane et al. (17) described how mismatches between farmers' attitudes and welfare strategies can result in non-compliance or even adversely effect animal welfare. In light of their finding, perhaps more in-depth examination of welfare practices on Irish dairy farms could be conducted in future research, ideally through interviews with farmers. This has been done previously in other countries, for example in Horseman et al. (42), investigating lameness in dairy cattle using interviews with farmers in the United Kingdom, and in Tucker et al. (43), where NZ dairy farmers identified a range of welfare issues. While recent research has examined public perceptions of animal welfare and dairy calf rearing (44), there is a dearth of studies examining farmers' views on the specific issue of male dairy calf welfare.

### Greater Integration

This section encompasses the responses related to beef merit and farm type in the results. While only a small number of farmers specifically made reference to the difficulty of reconciling the two sectors, a divide between beef and dairy farmers would be a significant obstacle to any measures aiming to develop greater dairy-beef integration. In terms of reducing the number of unwanted male dairy calves, there were many encouraging qualitative responses; such as those farmers who indicated a willingness to use sexed semen, especially if it was made more effective in terms of conception rates and accessible in terms of cost, and those farmers showing an awareness of the need to improve the beef merit of dairy animals, even if this would adversely impact milk production. These developments, if introduced, would still take several years to have a significant impact on the large number of male dairy calves in Ireland. While the benefits of using sexed semen have long been recognized (45), sexed semen usage has not yet become widespread. De Vries et al. (46) had anticipated that widespread usage would become common over the following decade, but this has not materialized as envisaged. In the interim, alternative ways for managing male dairy calves need to be considered. While the possibility of dairy farmers rearing male dairy calves for beef production on their own farms was not viewed as an effective option in the survey, one quarter of the dairy farmers still indicated that they currently rear male dairy calves for beef on their own farms.

Improving the beef merit of calves from the dairy herd in order to improve suitability for beef production, identified as a potential solution in New Zealand (47), will be an important step for greater dairy beef integration. Given the potential negative health and behavioral impact of transporting animals long distances to veal farm (48), alternative calf management strategies which allow for male dairy calves to be raised for beef in Ireland would have welfare benefits. A solution may have emerged from the free text question, where many farmers identified the potentially important role for other farm enterprise types, particularly suckler farmers, in rearing male dairy calves for beef production. However, the issues of who bears the risk and costs associated with greater integration will have to be carefully considered, as demonstrated in the following quote from a dairy farmer “*Presumably the dairy farmer bears the risk on the bull calf right through to slaughter or sale. Making this a mandatory requirement would be a massive shift in the system design so that the whole industry is mobilized. It's full Integration of the dairy/beef system in Ireland*.”

It is worth acknowledging the crucial value of the information obtained from these farmers' opinions on male dairy calves, which may help inform future policy solutions. Following the analysis carried out on the results of this survey, and discussions with the relevant stakeholders as part of the calf stakeholder group (comprising the main governmental, semi-state, farmer representative, and private organizations from across the Irish agricultural sector), it is clear that the barriers to better dairy-beef integration need to be examined in greater detail. This study has focused almost entirely on a sample of Irish dairy farmers. In this regard, the next logical step is to examine the views of a large sample of Irish beef farmers on potential solutions to the issue of the large number of male dairy calves in Ireland, to ensure that their unique perspectives are adequately considered before considering any changes to existing policy. Further qualitative research, such as interviews, with dairy farmers examining the themes arising from this study in greater detail may also provide further insight into these issues.

## Conclusions

To the knowledge of the authors, this is one of the first times Irish farmers have been surveyed on the welfare of male dairy calves. The estimated response rate of 30% is high for studies of this kind and indicates a high level of engagement. The respondents recognized that the primary responsibility for the welfare of dairy calves rested with the farmers themselves. While live exports were considered the main outlet for such calves, the wider use of sexed semen to ensure cross bred calves were female and the use of sires with more beefy characteristics were considered as potential solutions. However, poor beef price was considered a contributory factor which impacted disproportionately on dairy breed beef production.

## Data Availability Statement

The original contributions presented in the study are included in the article/Supplementary Material, further inquiries can be directed to the corresponding author/s.

## Ethics Statement

Ethical review and approval was not required for the study on human participants in accordance with the local legislation and institutional requirements. The participants provided their written informed consent to participate in this study.

## Author Contributions

RD conceived the idea of the paper, suggested questions for the survey, and provided observations on the results. MB reviewed the survey questions and provided observations on the results. AC and AB contributed to the design of the survey, redrafted the report of the results, and assisted with the literature review. JM assisted with the study design, analyzed the quantitative and qualitative data, presented the results to the calf stakeholder forum, and drafted this manuscript. DB oversaw the entire project and contributed to each stage from survey design through to the drafting of this manuscript. All authors read and approved the final manuscript.

## Conflict of Interest

The authors declare that the research was conducted in the absence of any commercial or financial relationships that could be construed as a potential conflict of interest.

## Supplementary Material

Additional File 1 Survey questions.

Additional File 2 Summary data for quantitative responses.
