# Supplementary material for: Exploring the Opinions of Irish Dairy Farmers Regarding Male Dairy Calves
Source: Front Vet Sci. 2021 Apr 20;8:635565. doi: 10.3389/fvets.2021.635565 (PMC8093389; doi:10.3389/fvets.2021.635565)
Supplement: Additional File 1 — Survey questions. [file Data_Sheet_1.docx]

**Additional file 1: Survey questions**

1. *Do you consent to participate in this study?* Yes; No.
2. *Farm type?* Dairy; Beef; Beef and Dairy; Other (please specify).
3. *Age?* 18-24; 25-34; 35-44; 45-54; 55-64; 65+.
4. *Which province do you live in?* Leinster; Munster; Connacht; Ulster.
5. *Do you farm on a full time or part time basis?* Full time; Part time.
6. *Are you concerned by the increased number of male dairy calves in recent years?* Yes; No.
7. *Please rank the following potential drivers for the increased number of male dairy calves in recent years, with 1 being the main causal factor.* Milk quotas were abolished; Profitability of dairy versus other farm types; Guidance from farm advisors; Increased demand for dairy products; Cooperatives; Farm press; DAFM policy/strategy.
8. *Please rank the following possible options in order of how effective you think they would be in managing the number of male dairy calves, with 1 being the most effective.* Increase exports of male dairy calves; Encourage greater use of sexed semen; Increase beef merit of male dairy calves; Try to establish a veal industry in Ireland; Greater use of contract rearing; Change DAFM policy; Rear male dairy calves for beef; Curtail production/reintroduce quotas.
9. *If live exports were to cease, please rank the following possible options in order of how effective you think they would be in managing the number of male dairy calves, with 1 being the most effective.* Encourage greater use of sexed semen; Increase beef merit of male dairy calves; Try to establish a veal industry in Ireland; Greater use of contract rearing; Change DAFM policy; Rear male dairy calves for beef; Curtail production/reintroduce quotas.
10. *Please select all stakeholders who you think are responsible for making changes to the number of male dairy calves.* Individual farmer; Teagasc; DAFM; Bord Bia; Farmer representatives; EU Commission.
11. *How many hours do you typically work on a spring day?* 0-6; 6-9; 9-12; 12-15; Over 15.
12. *What kind of assistance do you have on your farm? Please select all that apply*. Full time help from family; Full time paid labour; Part time help from family; Part time paid labour; No assistance.
13. *How many dairy cows do you have on your farm?* 0; 1-50; 50-100; 100-200; 200-500; Over 500.
14. *What do you consider the main role of your male dairy calves to be?* A means to get cows to produce milk; A product worthy of selling in its own right.
15. *What breed(s) are your dairy cows? Please select all that apply*. Friesian; Holstein; Cross-bred (e.g. Jersey cross); Jersey; Other.
16. *How do you currently manage your male dairy calf stock? Please select all apply.* Sell the calves via a mart; Sell the calves direct to a dealer/exporter; Raise for beef production on your farm; Contract rearing; Other (please specify).
17. *Have you enough calf accommodation on your farm?* Yes; No.
18. *To what extent do you agree with this statement - 'I am willing to pay for contract rearing for my male dairy calves'?* Strongly agree; Agree, Somewhat Agree; Somewhat Disagree; Disagree; Strongly disagree.
19. *Please provide any additional comments or suggestions you may have in the box below.*
