# Supplementary material for: Exploring the Opinions of Irish Dairy Farmers Regarding Male Dairy Calves
Source: Front Vet Sci. 2021 Apr 20;8:635565. doi: 10.3389/fvets.2021.635565 (PMC8093389; doi:10.3389/fvets.2021.635565)
Supplement: Additional File 2 — Summary data for quantitative responses. [file Data_Sheet_2.pdf]

Q1 Do you consent to participate in this study?

Answered: 879 Skipped: 0

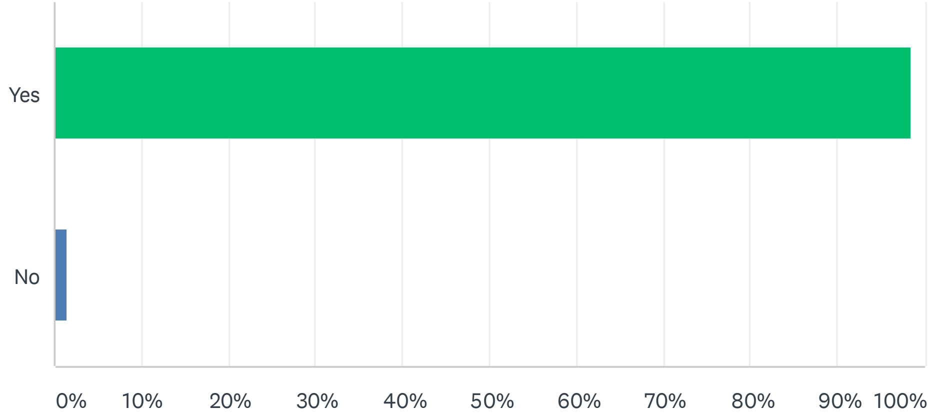

ANSWER CHOICES	RESPONSES	
Yes (1)	98.63%	867
No (2)	1.37%	12
TOTAL		879

BASIC STATISTICS				
Minimum	Maximum	Median	Mean	Standard Deviation
1.00	2.00	1.00	1.01	0.12

Q2 Farm type

Answered: 863 Skipped: 16

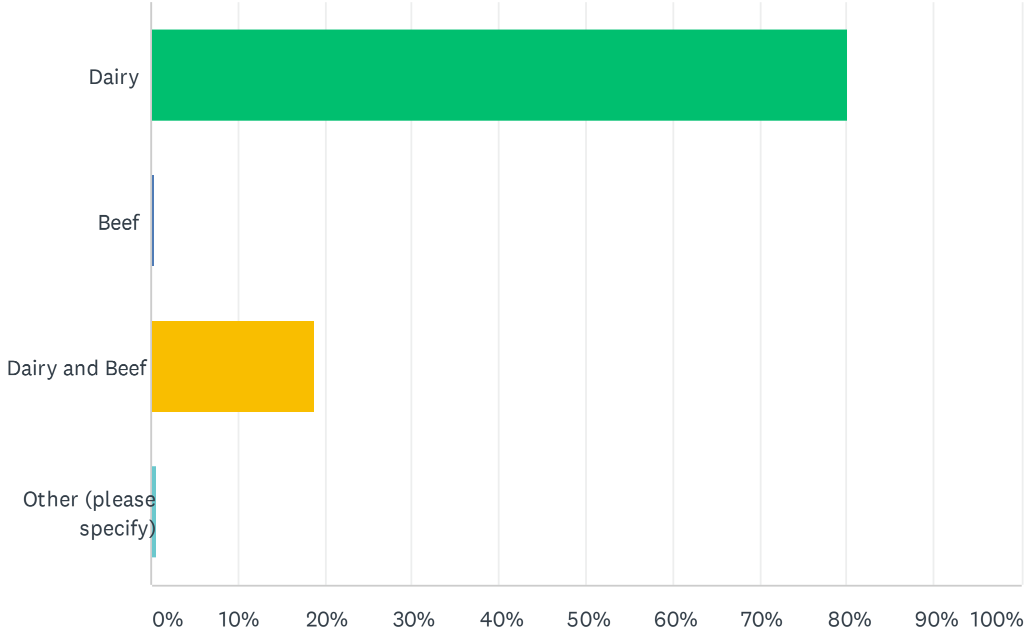

ANSWER CHOICES	RESPONSES	
Dairy	80.07%	691
Beef	0.46%	4
Dairy and Beef	18.89%	163
Other (please specify)	0.58%	5
TOTAL		863

BASIC STATISTICS				
Minimum	Maximum	Median	Mean	Standard Deviation
3.00	6.00	3.00	3.40	0.81

Q3 Age

Answered: 863 Skipped: 16

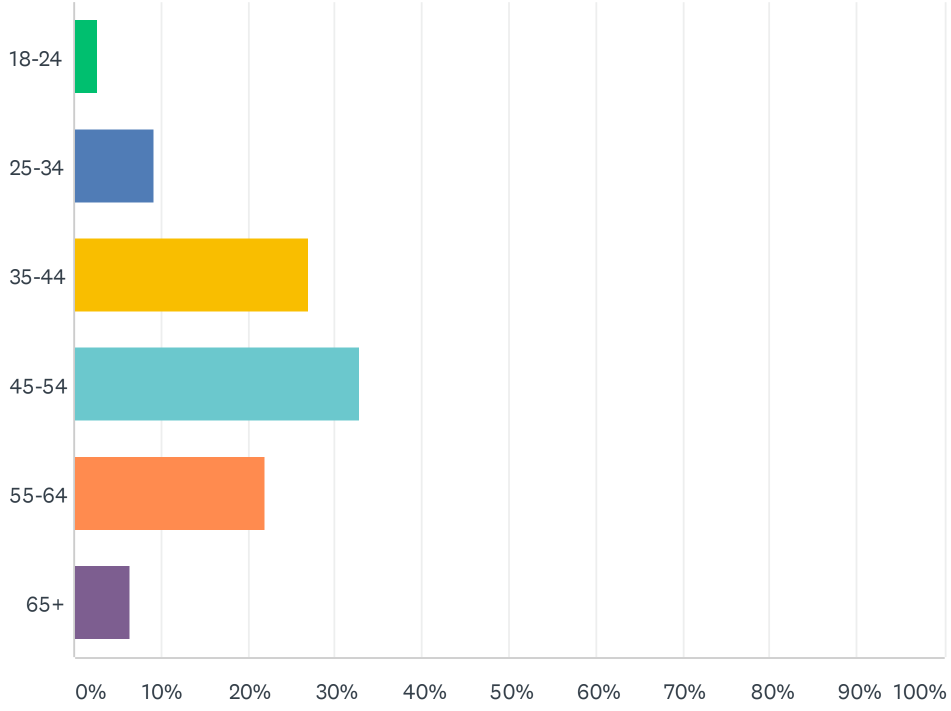

ANSWER CHOICES	RESPONSES	
18-24 (1)	2.67%	23
25-34 (2)	9.15%	79
35-44 (3)	27.00%	233
45-54 (4)	32.79%	283
55-64 (5)	21.90%	189
65+ (6)	6.49%	56
TOTAL		863

BASIC STATISTICS				
Minimum	Maximum	Median	Mean	Standard Deviation
1.00	6.00	4.00	3.82	1.15

Q4 Which province do you live in?

Answered: 863 Skipped: 16

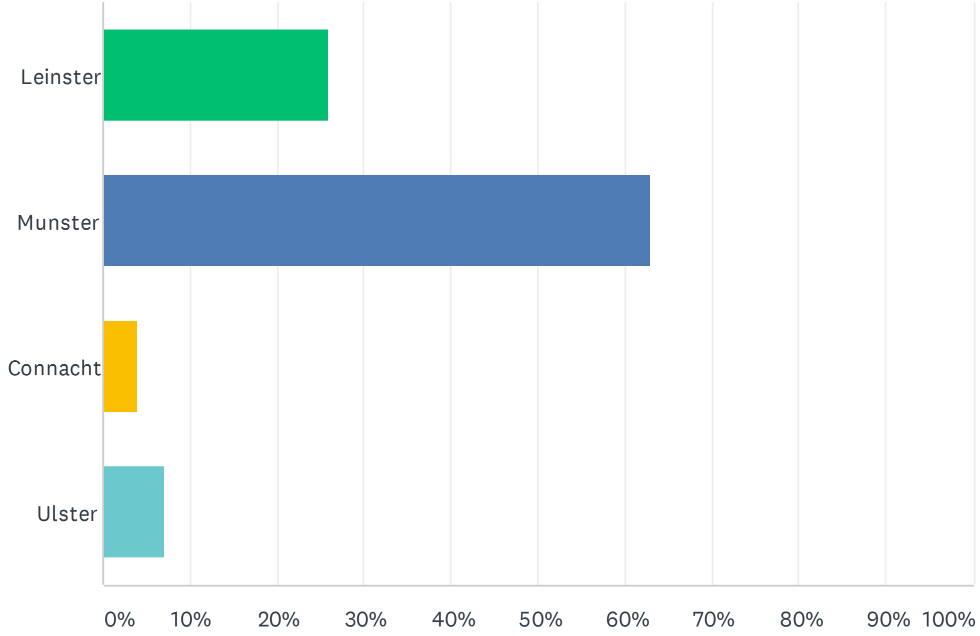

ANSWER CHOICES	RESPONSES	
Leinster (1)	25.84%	223
Munster (2)	63.04%	544
Connacht (3)	4.06%	35
Ulster (4)	7.07%	61
TOTAL		863

BASIC STATISTICS				
Minimum	Maximum	Median	Mean	Standard Deviation
1.00	4.00	2.00	1.92	0.76

Q5 Do you farm on a full time or part time basis?

Answered: 863 Skipped: 16

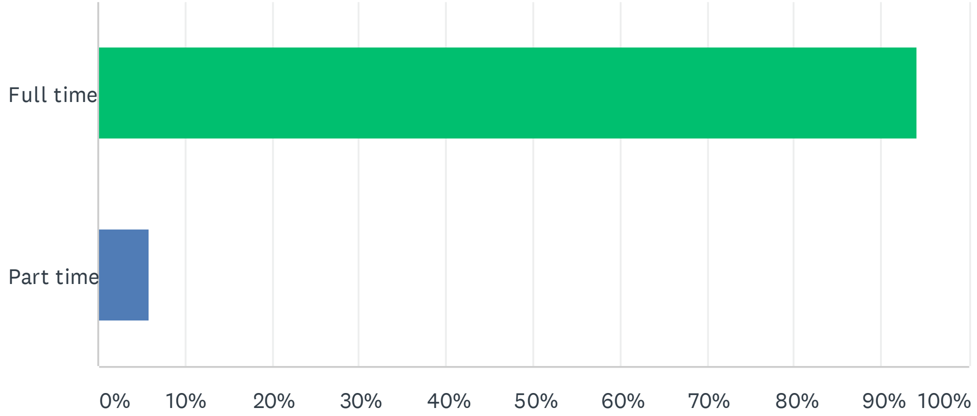

ANSWER CHOICES	RESPONSES	
Full time (1)	94.09%	812
Part time (2)	5.91%	51
TOTAL		863

BASIC STATISTICS				
Minimum	Maximum	Median	Mean	Standard Deviation
1.00	2.00	1.00	1.06	0.24

Q6 Are you concerned by the increased number of male dairy calves in recent years?

Answered: 681   Skipped: 198

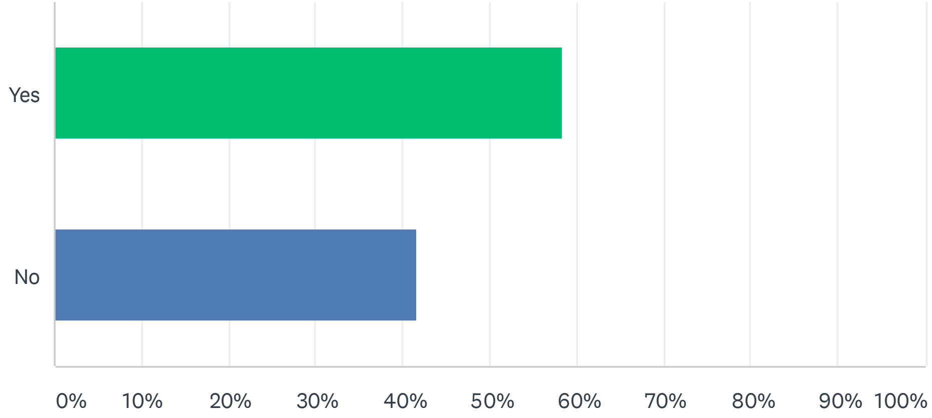

ANSWER CHOICES	RESPONSES	
Yes (1)	58.30%	397
No (2)	41.70%	284
TOTAL		681

BASIC STATISTICS				
Minimum	Maximum	Median	Mean	Standard Deviation
1.00	2.00	1.00	1.42	0.49

Q7 Please rank the following potential drivers for the increased number of male dairy calves in recent years, with 1 being the main causal factor.

Answered: 681 Skipped: 198

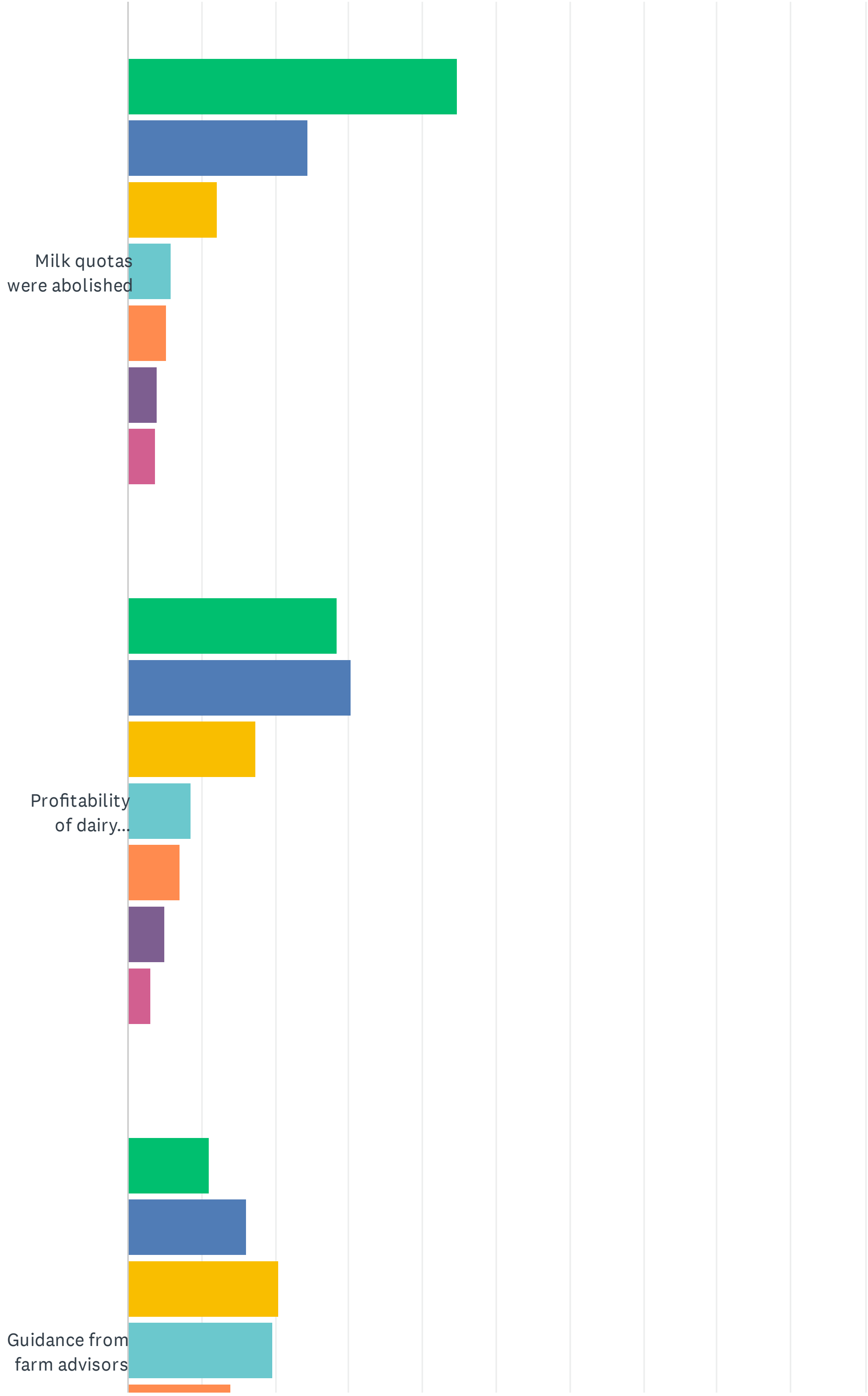

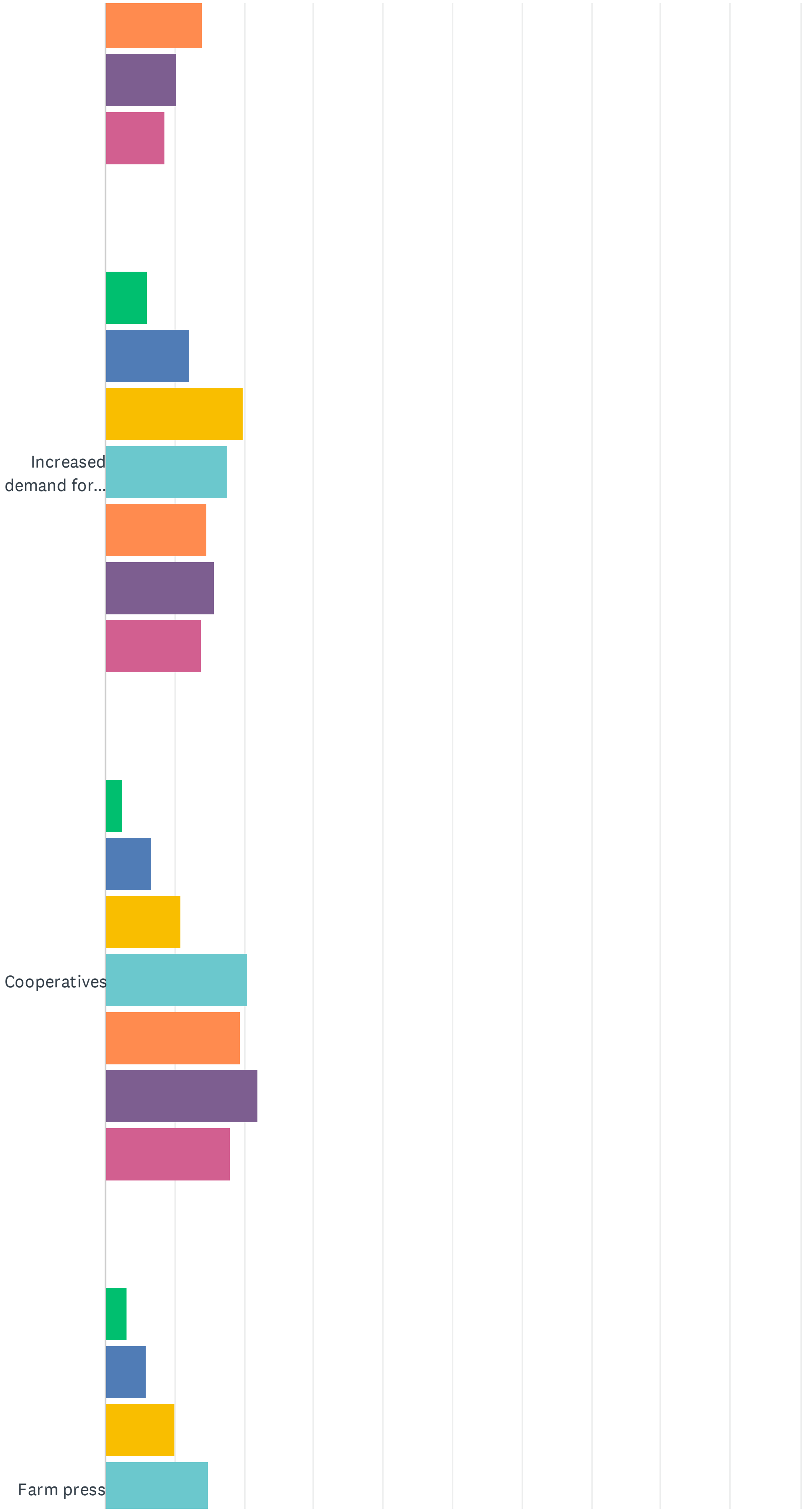
Increased demand for...
Cooperatives
Farm press

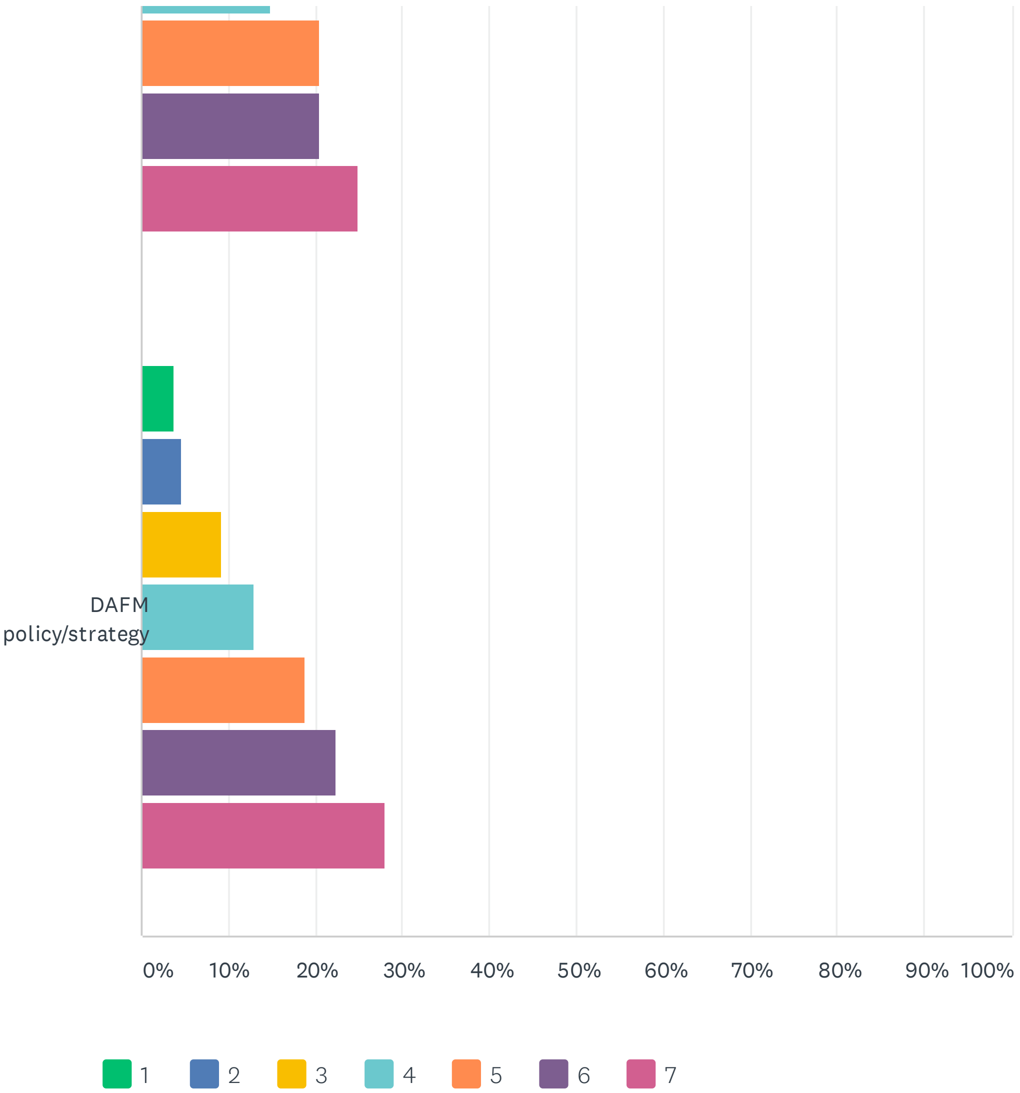

	1	2	3	4	5	6	7	TOTAL	SCORE
Milk quotas were abolished	44.79% 305	24.38% 166	12.04% 82	5.87% 40	5.29% 36	3.96% 27	3.67% 25	681	5.71
Profitability of dairy farming compared to other farm types	28.49% 194	30.40% 207	17.33% 118	8.52% 58	7.20% 49	4.99% 34	3.08% 21	681	5.37
Guidance from farm advisors	11.01% 75	16.01% 109	20.41% 139	19.68% 134	14.10% 96	10.28% 70	8.52% 58	681	4.25
Increased demand for dairy products	6.17% 42	12.04% 82	19.97% 136	17.62% 120	14.68% 100	15.71% 107	13.80% 94	681	3.75
Cooperatives	2.50% 17	6.61% 45	10.87% 74	20.56% 140	19.38% 132	22.03% 150	18.06% 123	681	3.14
Farm press	3.23% 22	5.87% 40	10.13% 69	14.83% 101	20.56% 140	20.56% 140	24.82% 169	681	2.95
DAFM policy/strategy	3.82% 26	4.70% 32	9.25% 63	12.92% 88	18.80% 128	22.47% 153	28.05% 191	681	2.82

BASIC STATISTICS					
	**MINIMUM**	**MAXIMUM**	**MEDIAN**	**MEAN**	**STANDARD DEVIATION**
Milk quotas were abolished	1.00	7.00	2.00	2.29	1.66
DAFM policy/strategy	1.00	7.00	6.00	5.18	1.69
Profitability of dairy farming compared to other farm types	1.00	7.00	2.00	2.63	1.62
Increased demand for dairy products	1.00	7.00	4.00	4.25	1.79
Guidance from farm advisors	1.00	7.00	4.00	3.75	1.76
Farm press	1.00	7.00	5.00	5.05	1.67
Cooperatives	1.00	7.00	5.00	4.86	1.60

Q8 Please rank the following possible options in order of how effective you think they would be in managing the number of male dairy calves, with 1 being the most effective.

Answered: 681 Skipped: 198

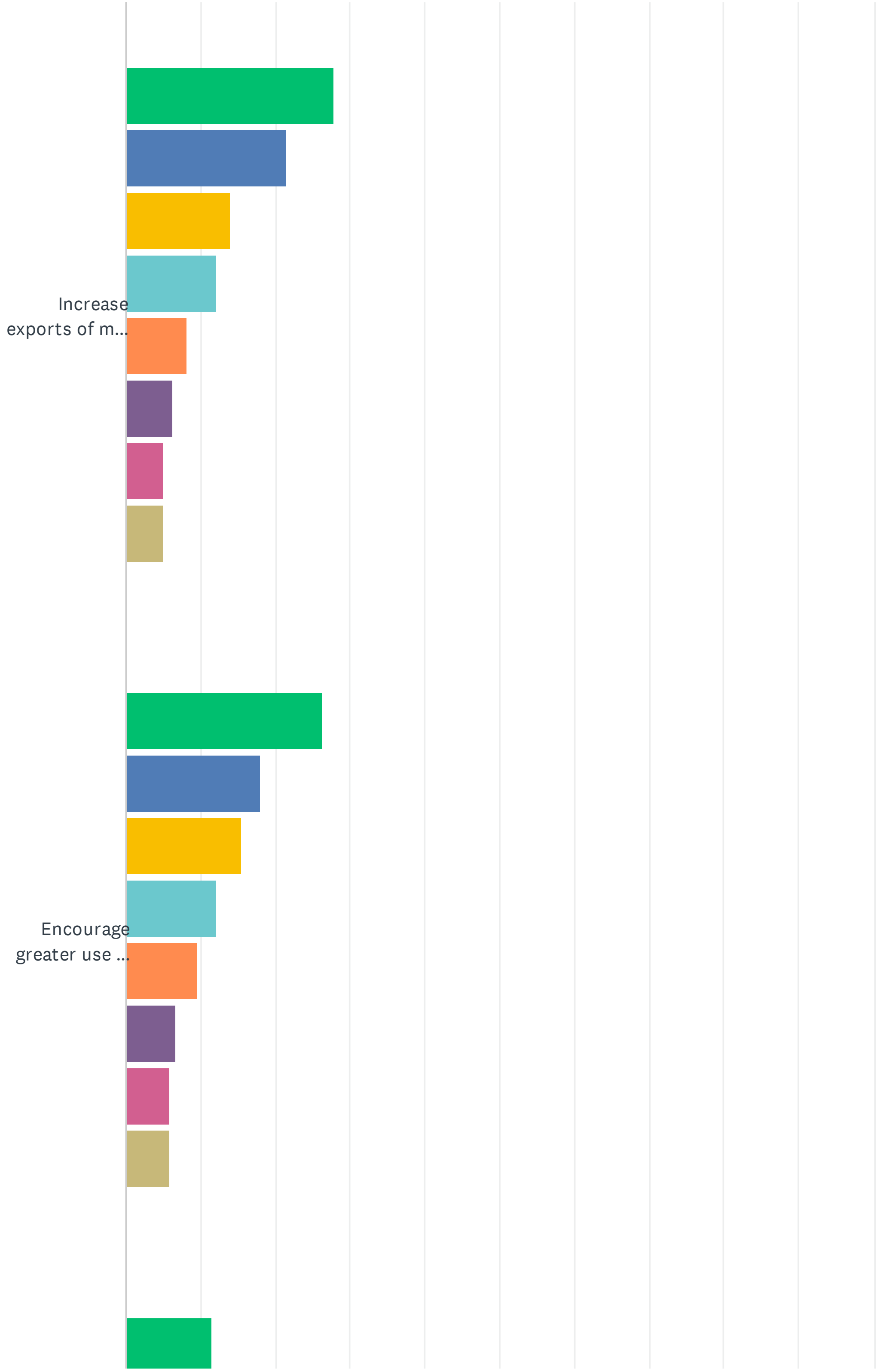

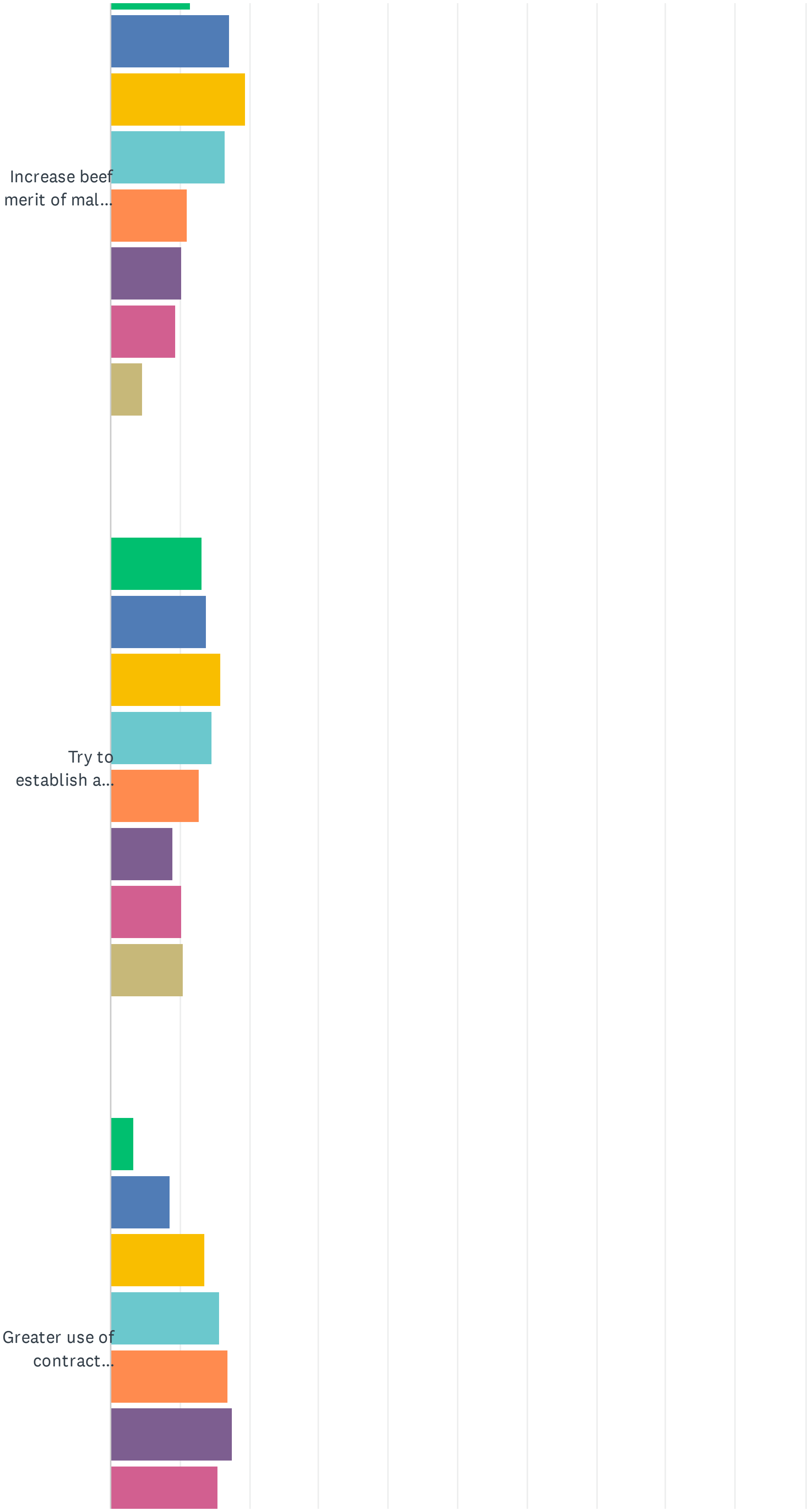
Increase beef merit of mal...
Try to establish a...
Greater use of contract...

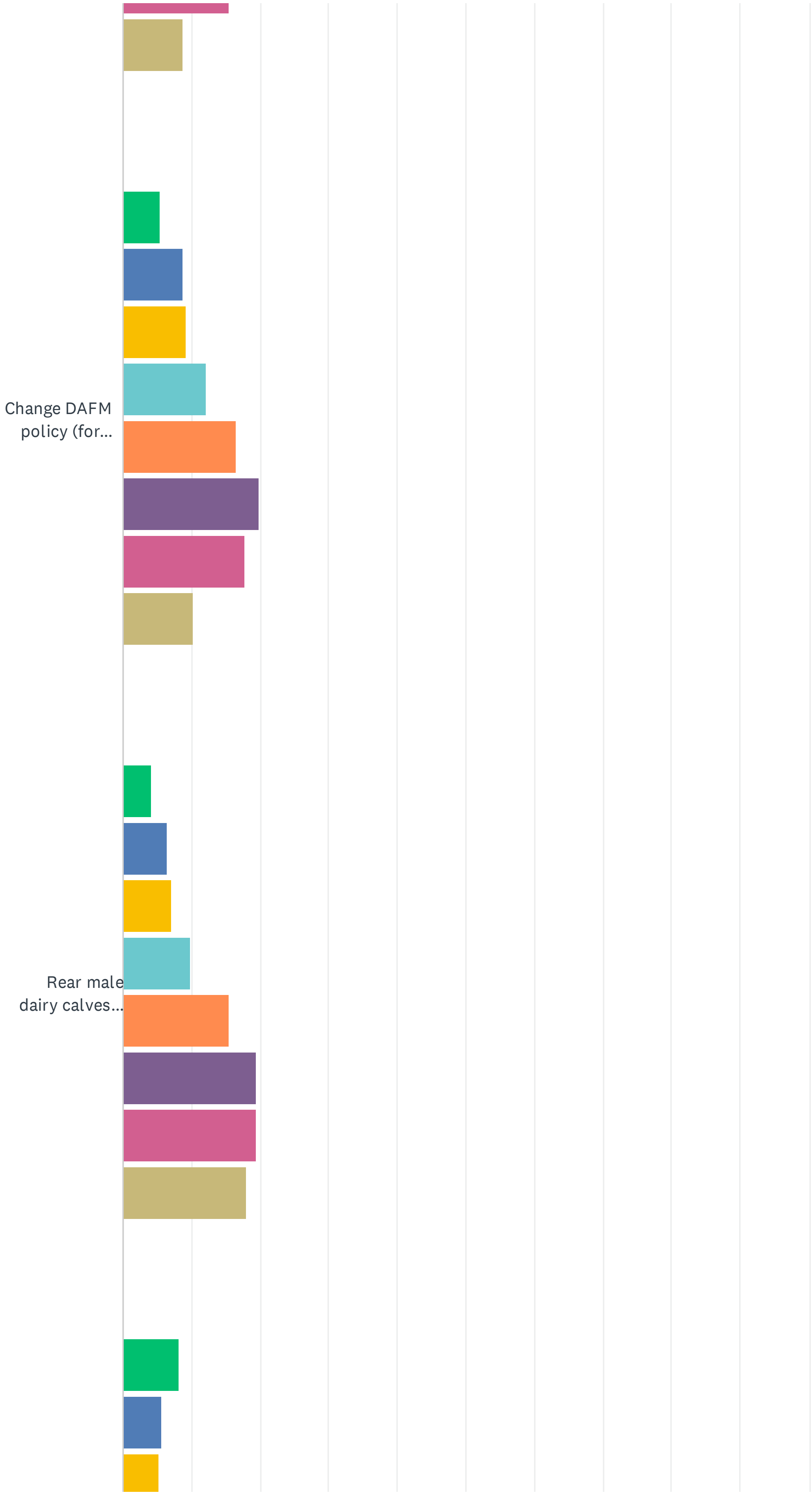
Change DAFM
policy (for...
Rear male
dairy calves...

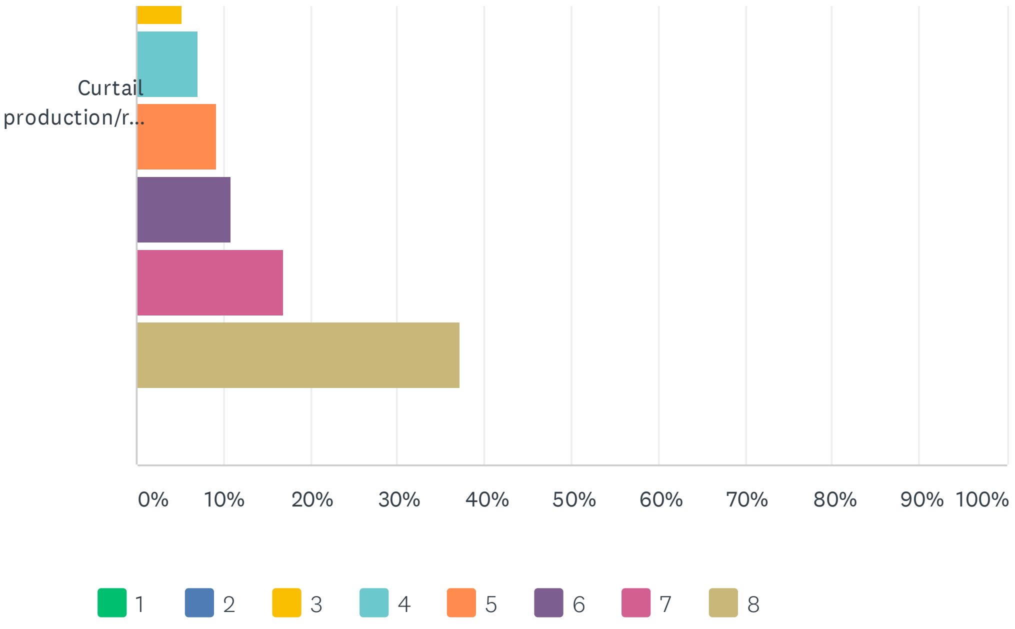

	1	2	3	4	5	6	7	8	TOTAL	SCORE
Increase exports of male dairy calves	27.75% 189	21.59% 147	14.10% 96	12.04% 82	8.22% 56	6.31% 43	4.99% 34	4.99% 34	681	5.85
Encourage greater use of sexed semen	26.43% 180	17.91% 122	15.42% 105	12.04% 82	9.69% 66	6.75% 46	5.87% 40	5.87% 40	681	5.66
Increase beef merit of male dairy calves	11.45% 78	17.18% 117	19.53% 133	16.45% 112	11.16% 76	10.28% 70	9.40% 64	4.55% 31	681	5.10
Try to establish a veal industry in Ireland	13.22% 90	13.80% 94	15.86% 108	14.68% 100	12.78% 87	8.96% 61	10.28% 70	10.43% 71	681	4.80
Greater use of contract rearing for male dairy calves	3.38% 23	8.66% 59	13.51% 92	15.71% 107	17.03% 116	17.47% 119	15.42% 105	8.81% 60	681	4.07
Change DAFM policy (for example, encourage farm types other than dairy)	5.43% 37	8.81% 60	9.10% 62	12.19% 83	16.59% 113	19.82% 135	17.77% 121	10.28% 70	681	3.92
Rear male dairy calves for beef production on own farm	4.26% 29	6.46% 44	7.20% 49	9.84% 67	15.42% 105	19.53% 133	19.38% 132	17.91% 122	681	3.49
Curtail production/re-introduce quotas	8.08% 55	5.58% 38	5.29% 36	7.05% 48	9.10% 62	10.87% 74	16.89% 115	37.15% 253	681	3.11

BASIC STATISTICS

	MINIMUM	MAXIMUM	MEDIAN	MEAN	STANDARD DEVIATION
Curtail production/re-introduce quotas	1.00	8.00	7.00	5.89	2.32
Change DAFM policy (for example, encourage farm types other than dairy)	1.00	8.00	5.00	5.08	1.99
Increase beef merit of male dairy calves	1.00	8.00	4.00	3.90	2.00
Increase exports of male dairy calves	1.00	8.00	3.00	3.15	2.09
Try to establish a veal industry in Ireland	1.00	8.00	4.00	4.20	2.22
Encourage greater use of sexed semen	1.00	8.00	3.00	3.34	2.16
Rear male dairy calves for beef production on own farm	1.00	8.00	6.00	5.51	1.99
Greater use of contract rearing for male dairy calves	1.00	8.00	5.00	4.93	1.90

Q9 If live exports were to cease, please rank the following possible options in order of how effective you think they would be in managing the number of male dairy calves, with 1 being the most effective.

Answered: 681 Skipped: 198

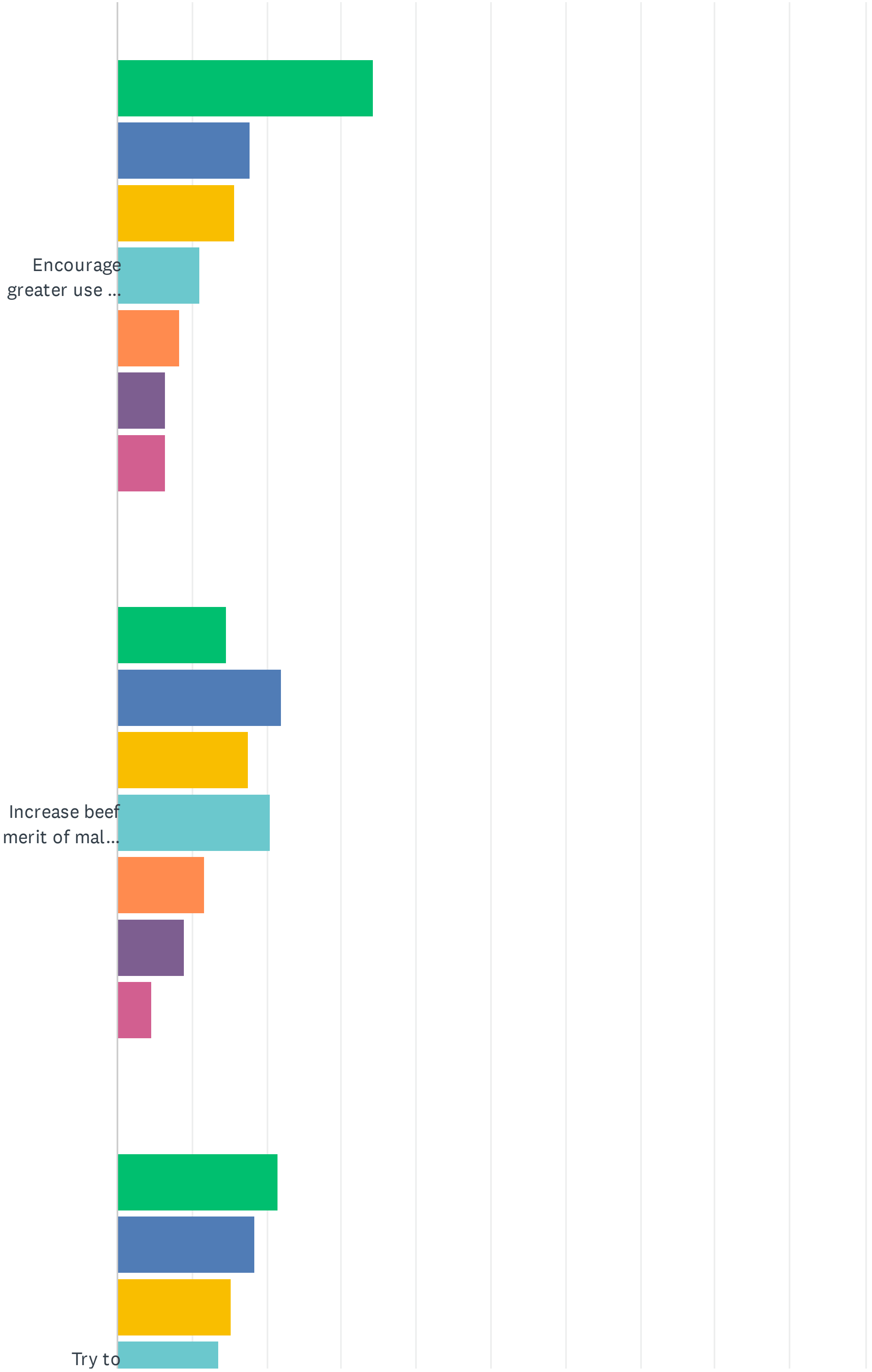

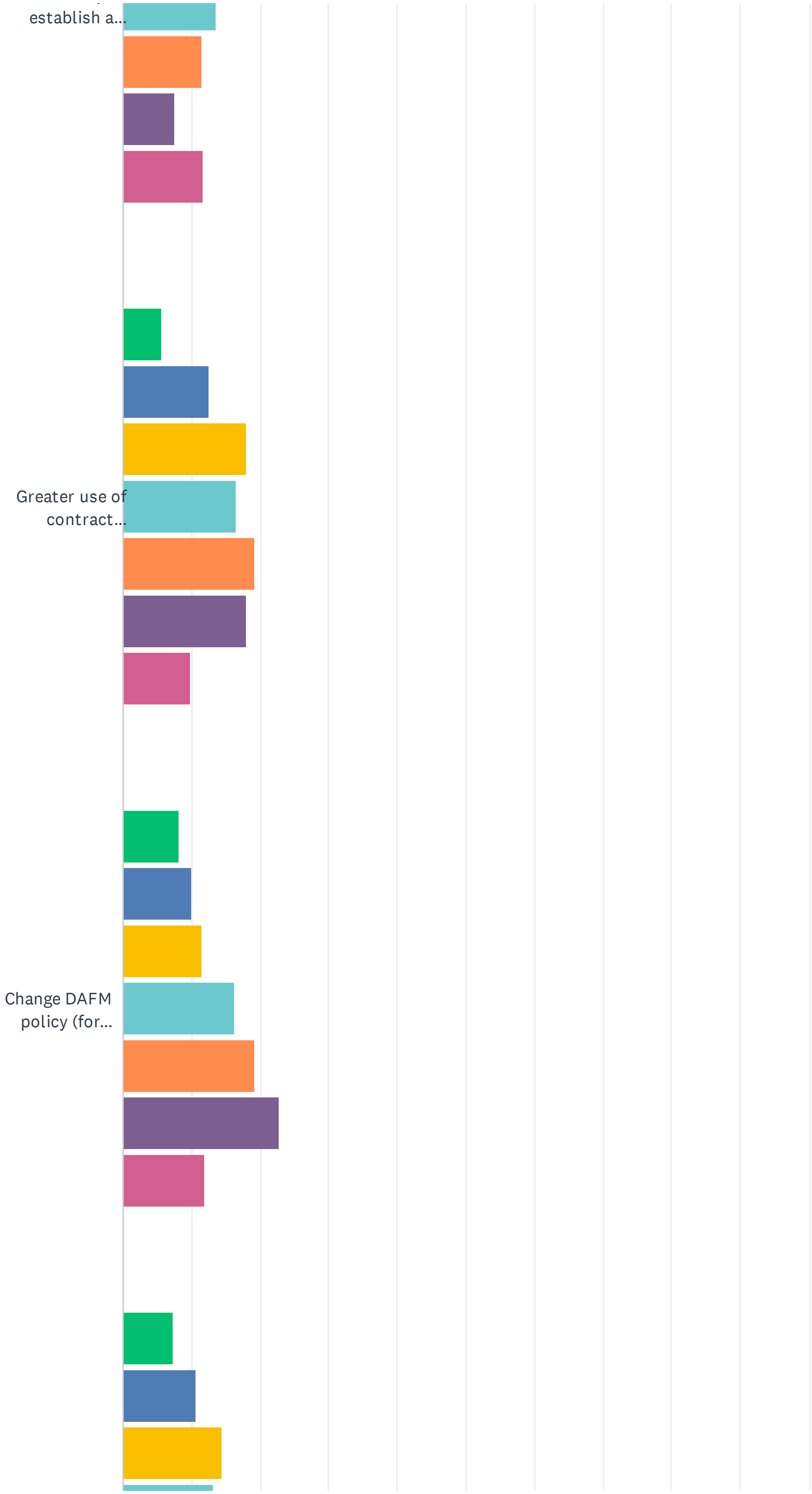
establish a...
Greater use of contract...
Change DAFM policy (for...

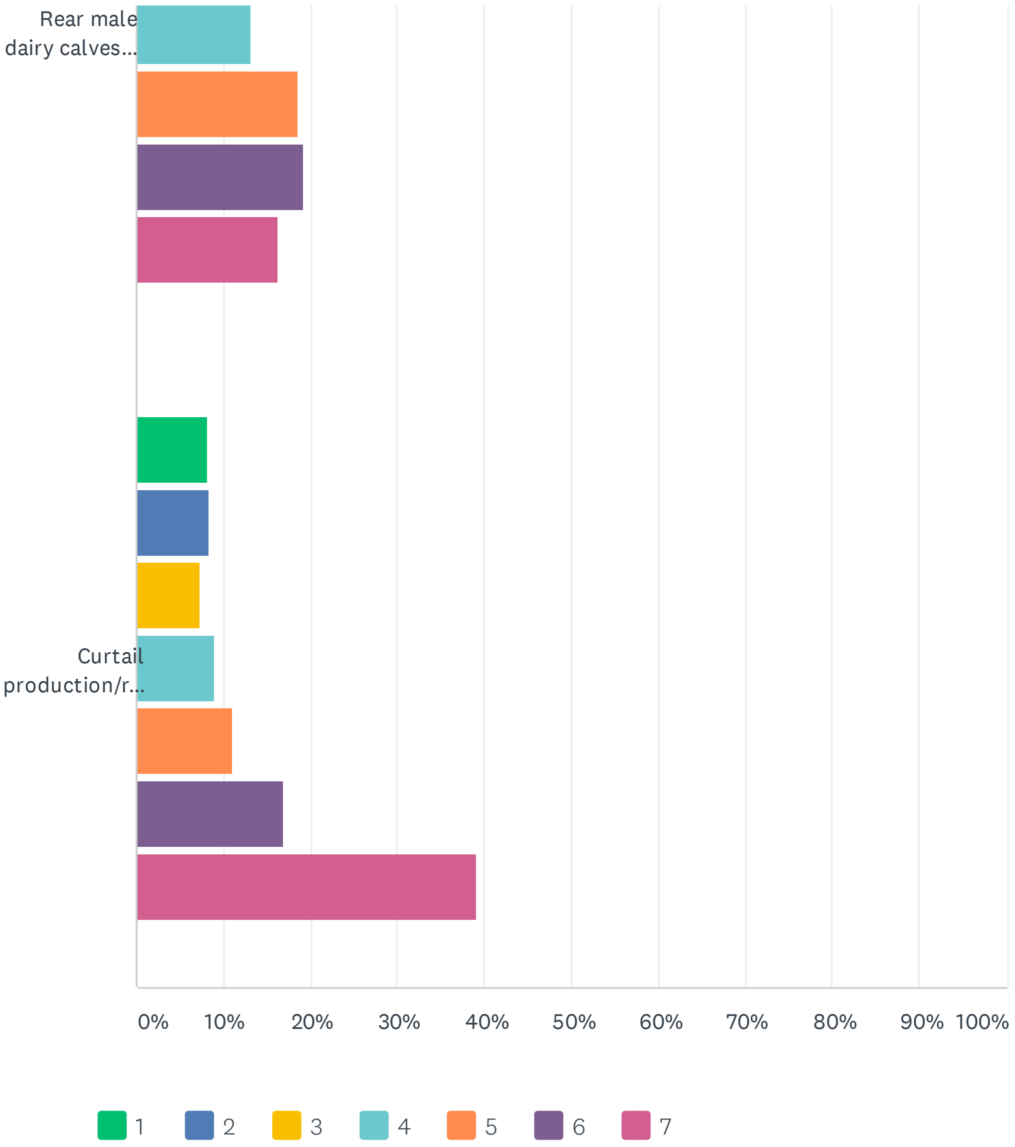

	1	2	3	4	5	6	7	TOTAL	SCORE
Encourage greater use of sexed semen	34.21%<br>233	17.77%<br>121	15.71%<br>107	11.01%<br>75	8.37%<br>57	6.46%<br>44	6.46%<br>44	681	5.13
Increase beef merit of male dairy calves	14.68%<br>100	21.88%<br>149	17.62%<br>120	20.41%<br>139	11.75%<br>80	8.96%<br>61	4.70%<br>32	681	4.62
Try to establish a veal industry in Ireland	21.59%<br>147	18.50%<br>126	15.27%<br>104	13.66%<br>93	11.60%<br>79	7.64%<br>52	11.75%<br>80	681	4.55
Greater use of contract rearing for male dairy calves	5.73%<br>39	12.63%<br>86	18.06%<br>123	16.45%<br>112	19.24%<br>131	18.06%<br>123	9.84%<br>67	681	3.76
Change DAFM policy (for example, encourage farm types other than dairy)	8.22%<br>56	10.13%<br>69	11.45%<br>78	16.30%<br>111	19.24%<br>131	22.76%<br>155	11.89%<br>81	681	3.56
Rear male dairy calves for beef production on own farm	7.34%<br>50	10.72%<br>73	14.54%<br>99	13.22%<br>90	18.65%<br>127	19.24%<br>131	16.30%<br>111	681	3.52
Curtail production/re-introduce quotas	8.22%<br>56	8.37%<br>57	7.34%<br>50	8.96%<br>61	11.16%<br>76	16.89%<br>115	39.06%<br>266	681	2.87

BASIC STATISTICS					
	MINIMUM	MAXIMUM	MEDIAN	MEAN	STANDARD DEVIATION
Rear male dairy calves for beef production on own farm	1.00	7.00	5.00	4.48	1.85
Try to establish a veal industry in Ireland	1.00	7.00	3.00	3.45	2.00
Encourage greater use of sexed semen	1.00	7.00	2.00	2.87	1.89
Increase beef merit of male dairy calves	1.00	7.00	3.00	3.38	1.70
Greater use of contract rearing for male dairy calves	1.00	7.00	4.00	4.24	1.72
Curtail production/re-introduce quotas	1.00	7.00	6.00	5.13	2.04
Change DAFM policy (for example, encourage farm types other than dairy)	1.00	7.00	5.00	4.44	1.80

Q10 Please select all stakeholders who you think are responsible for making changes to the number of male dairy calves.

Answered: 681 Skipped: 198

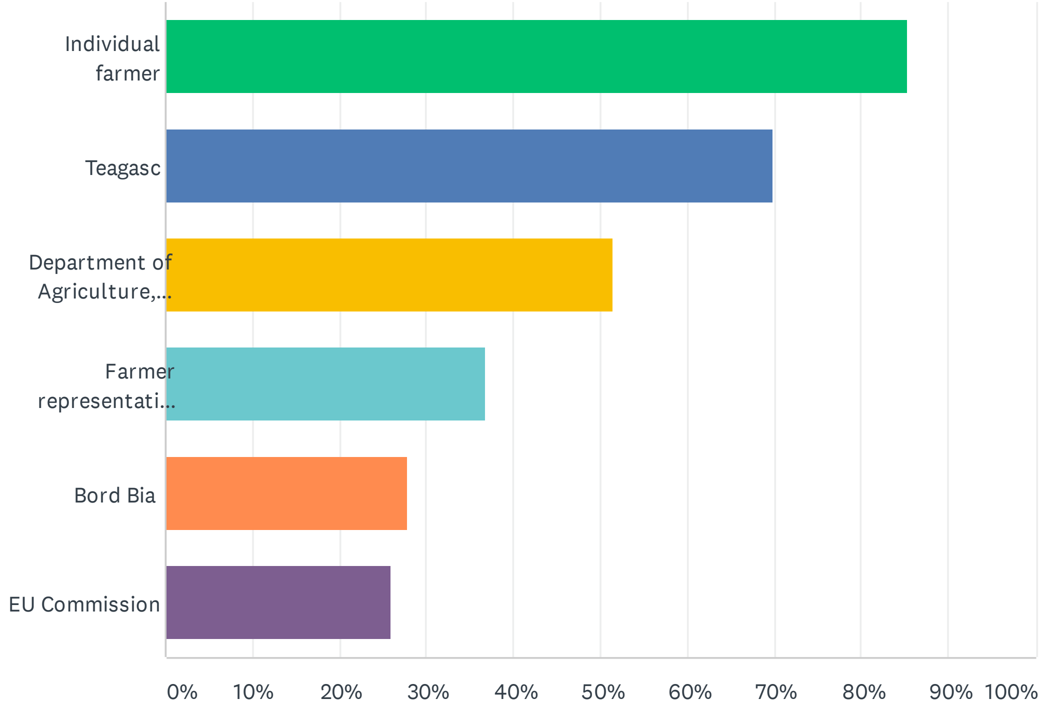

ANSWER CHOICES	RESPONSES	
Individual farmer (1)	85.32%	581
Teagasc (3)	69.90%	476
Department of Agriculture, Food and the Marine (2)	51.40%	350
Farmer representative organisations (6)	36.86%	251
Bord Bia (4)	27.75%	189
EU Commission (5)	25.99%	177
Total Respondents: 681		

BASIC STATISTICS				
Minimum	Maximum	Median	Mean	Standard Deviation
1.00	6.00	3.00	2.89	1.69

Q11 How many hours do you typically work on a spring day?

Answered: 681 Skipped: 198

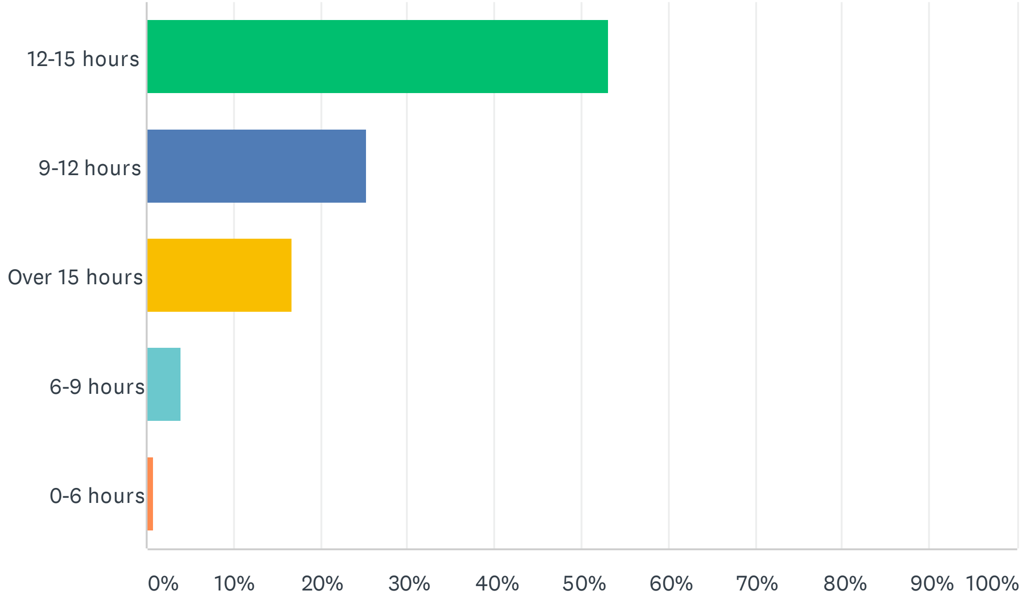

ANSWER CHOICES	RESPONSES	
12-15 hours (4)	53.16%	362
9-12 hours (3)	25.26%	172
Over 15 hours (5)	16.74%	114
6-9 hours (2)	3.96%	27
0-6 hours (1)	0.88%	6
TOTAL		681

BASIC STATISTICS				
Minimum	Maximum	Median	Mean	Standard Deviation
1.00	5.00	4.00	3.81	0.79

Q12 What kind of assistance do you have on your farm? Please select all that apply.

Answered: 681 Skipped: 198

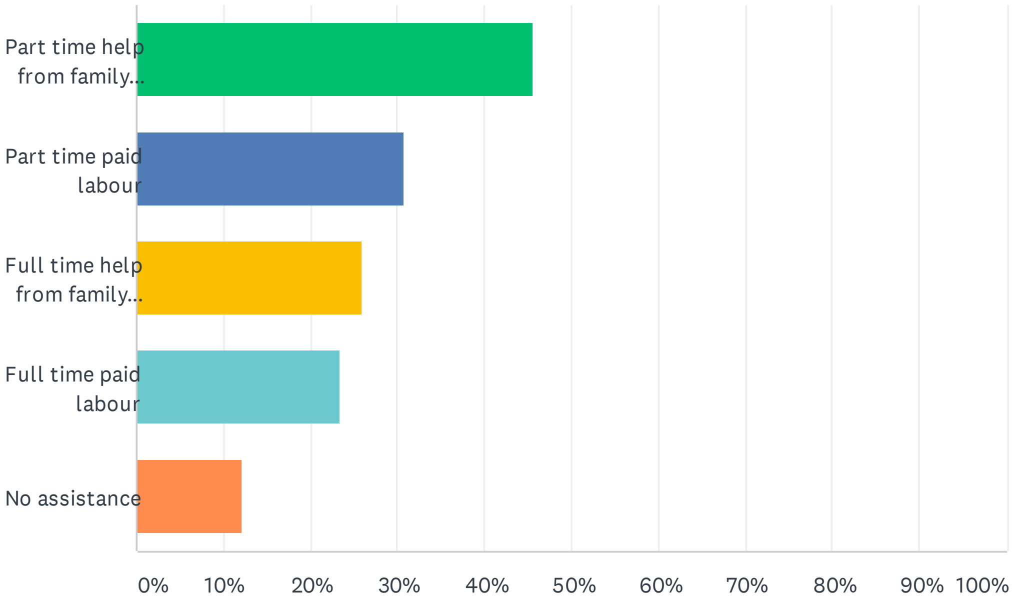

ANSWER CHOICES	RESPONSES	
Part time help from family member(s) (2)	45.52%	310
Part time paid labour (4)	30.69%	209
Full time help from family member(s) (1)	25.99%	177
Full time paid labour (3)	23.49%	160
No assistance (5)	12.19%	83
Total Respondents: 681		

BASIC STATISTICS

Minimum	Maximum	Median	Mean	Standard Deviation
1.00	5.00	2.00	2.69	1.25

Q13 How many dairy cows do you have on your farm?

Answered: 681   Skipped: 198

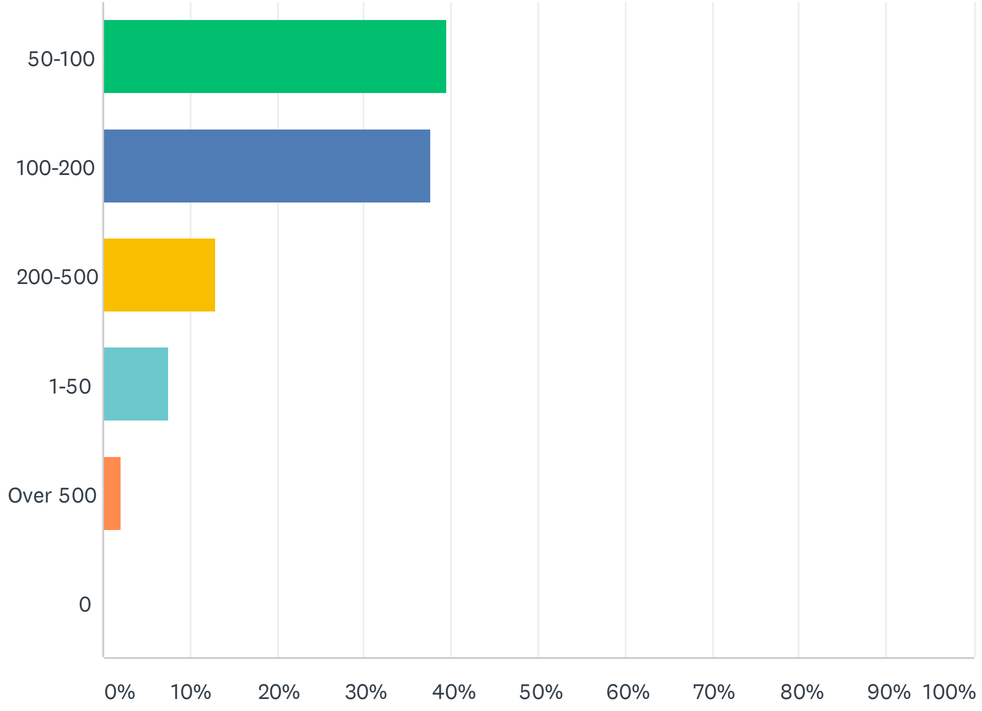

ANSWER CHOICES	RESPONSES	
50-100 (3)	39.50%	269
100-200 (4)	37.59%	256
200-500 (5)	12.92%	88
1-50 (2)	7.64%	52
Over 500 (6)	2.06%	14
0 (1)	0.29%	2
TOTAL		681

BASIC STATISTICS				
Minimum	Maximum	Median	Mean	Standard Deviation
1.00	6.00	4.00	3.61	0.89

Q14 What do you consider the main role of your male dairy calves to be?

Answered: 672 Skipped: 207

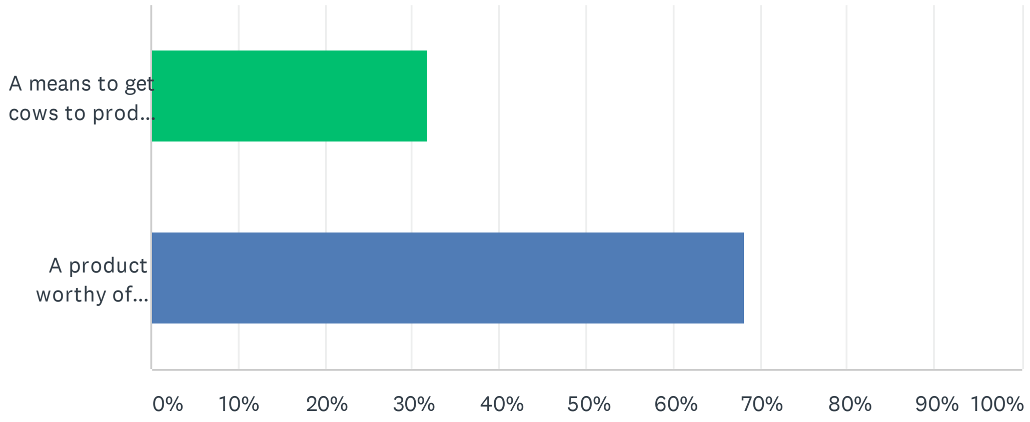

ANSWER CHOICES	RESPONSES	
A means to get cows to produce milk (1)	31.85%	214
A product worthy of selling in its own right (2)	68.15%	458
TOTAL		672

BASIC STATISTICS				
Minimum	Maximum	Median	Mean	Standard Deviation
1.00	2.00	2.00	1.68	0.47

Q15 What breed(s) are your dairy cows? Please select all that apply.

Answered: 672 Skipped: 207

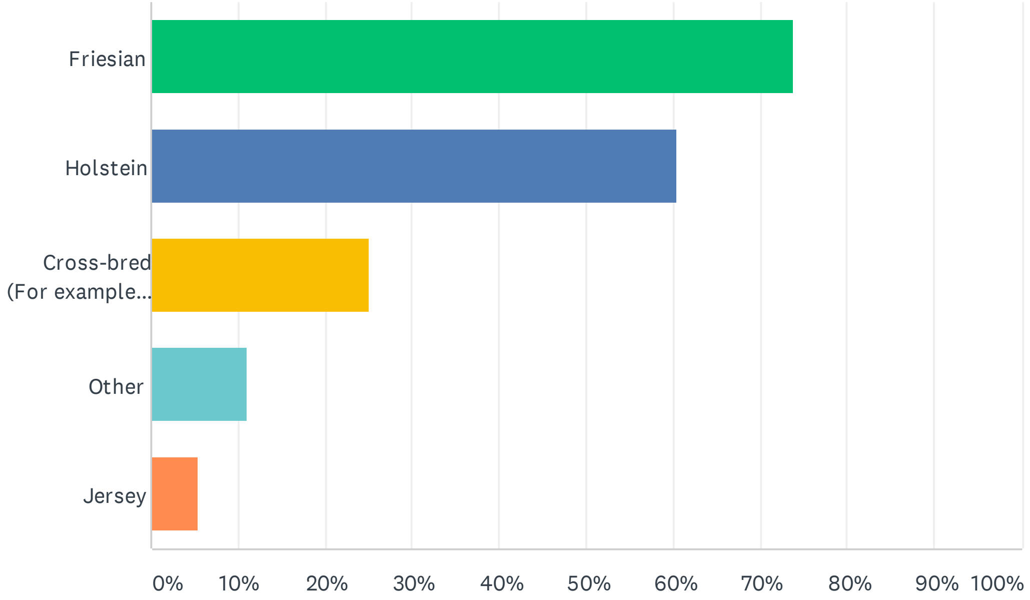

ANSWER CHOICES	RESPONSES	
Friesian (1)	73.81%	496
Holstein (2)	60.42%	406
Cross-bred (For example Jersey-Friesian cross) (4)	25.15%	169
Other (5)	11.16%	75
Jersey (3)	5.36%	36
Total Respondents: 672		

BASIC STATISTICS				
Minimum	Maximum	Median	Mean	Standard Deviation
1.00	5.00	2.00	2.09	1.26

Q16 How do you currently manage your male dairy calf stock? Please select all apply.

Answered: 672 Skipped: 207

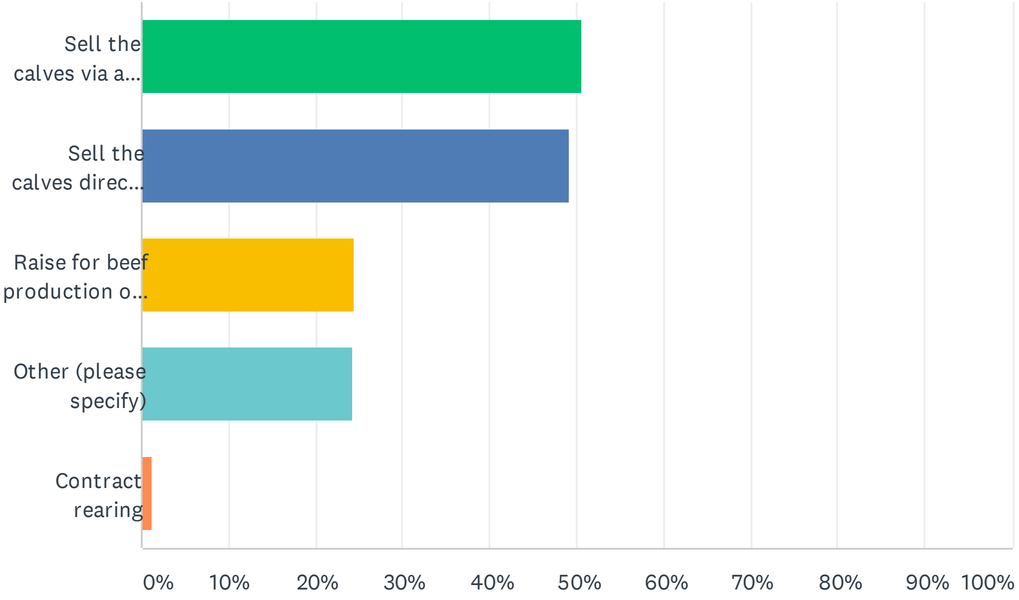

ANSWER CHOICES	RESPONSES	
Sell the calves via a mart (3)	50.60%	340
Sell the calves direct to a dealer/exporter (4)	49.11%	330
Raise for beef production on your farm (1)	24.40%	164
Other (please specify) (5)	24.26%	163
Contract rearing (2)	1.34%	9
Total Respondents: 672		

BASIC STATISTICS

Minimum	Maximum	Median	Mean	Standard Deviation
1.00	5.00	3.00	3.32	1.24

Q17 Have you enough calf accommodation on your farm?

Answered: 672 Skipped: 207

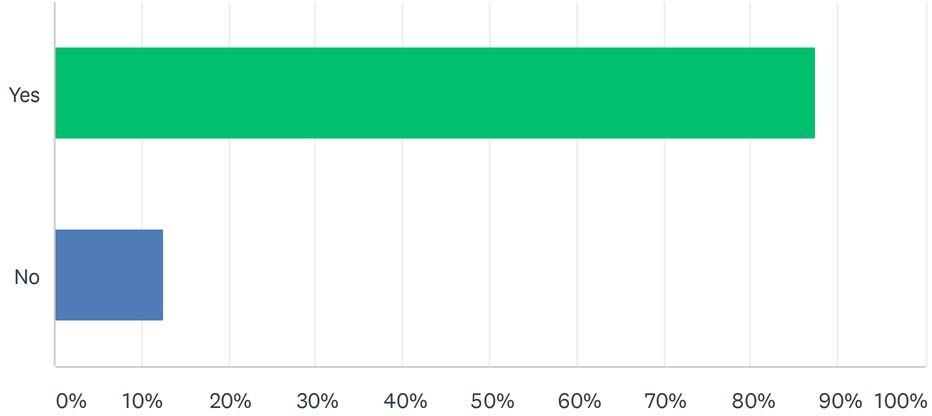

ANSWER CHOICES	RESPONSES	
Yes (1)	87.50%	588
No (2)	12.50%	84
TOTAL		672

BASIC STATISTICS				
Minimum	Maximum	Median	Mean	Standard Deviation
1.00	2.00	1.00	1.13	0.33

Q18 To what extent do you agree with this statement - 'I am willing to pay for contract rearing for my male dairy calves'

Answered: 672 Skipped: 207

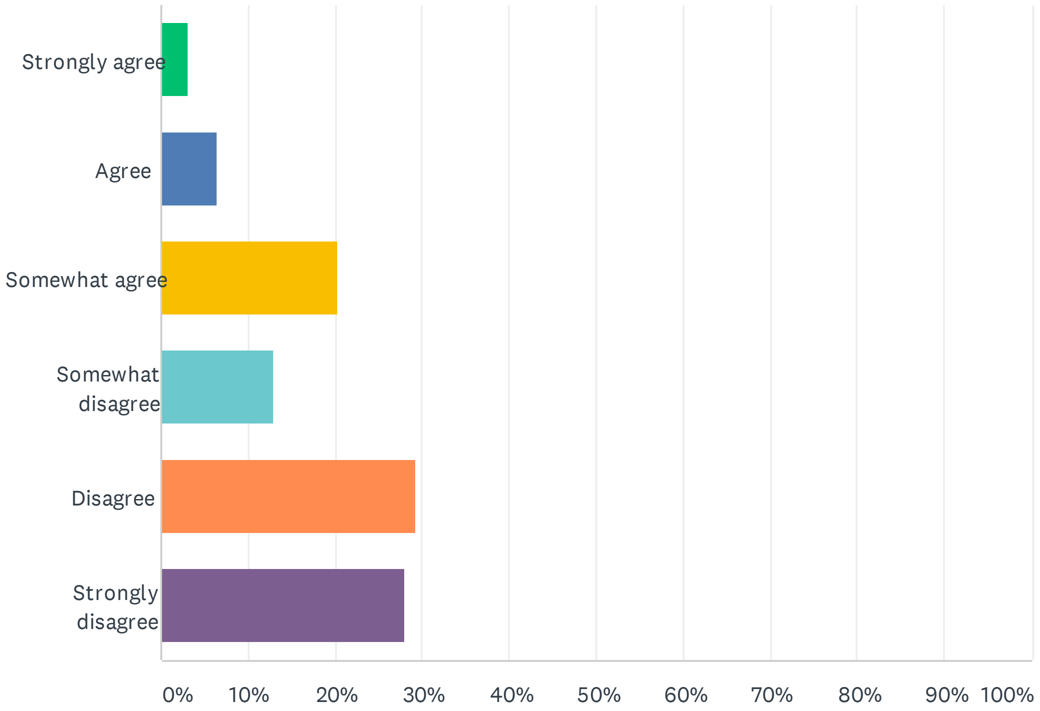

ANSWER CHOICES	RESPONSES	
Strongly agree (1)	3.13%	21
Agree (2)	6.40%	43
Somewhat agree (3)	20.24%	136
Somewhat disagree (4)	12.95%	87
Disagree (5)	29.32%	197
Strongly disagree (6)	27.98%	188
TOTAL		672

BASIC STATISTICS				
Minimum	Maximum	Median	Mean	Standard Deviation
1.00	6.00	5.00	4.43	1.40

Q19 Please provide any additional comments or suggestions you may have in the box below.

Answered: 402 Skipped: 477
